# Advancing CdSe quantum dots for batteries and supercapacitors: electrochemical frontiers

**DOI:** 10.1039/d5ra02414e

**Published:** 2025-05-14

**Authors:** Mosstafa Kazemi, Hadi Noorizadeh, Yashwantsinh Jadeja, Shelesh Krishna Saraswat, Rekha M. M., Aman Shankhyan, Supriya S., Kamal Kant Joshi

**Affiliations:** a Young Researchers and Elite Club, Tehran Branch, Islamic Azad University Tehran Iran hadinoorizadeh@yahoo.com; b Marwadi University Research Center, Department of Chemistry, Faculty of Science, Marwadi University Rajkot-360003 Gujarat India; c Department of ECE, GLA University Mathura-281406 INDIA; d Department of Chemistry and Biochemistry, School of Sciences, JAIN (Deemed to be University) Bangalore Karnataka India; e Centre for Research Impact & Outcome, Chitkara University Institute of Engineering and Technology, Chitkara University Rajpura 140401 Punjab India; f Department of Chemistry, Sathyabama Institute of Science and Technology Chennai Tamil Nadu India; g Department of Allied Science, Graphic Era Hill University Dehradun India; h Graphic Era Deemed to be University Dehradun Uttarakhand India

## Abstract

Cadmium selenide (CdSe) quantum dots (QDs) have emerged as transformative nanomaterials in energy storage, leveraging their size-tunable electronic properties and high surface area to push the boundaries of batteries and supercapacitors. This review marks the first dedicated investigation of CdSe QDs specifically tailored for batteries and supercapacitors unraveling their potential to enhance charge storage, cycling stability, and electrochemical efficiency. We highlight cutting-edge advancements in integrating CdSe QDs into lithium-ion batteries, lithium–oxygen batteries, and supercapacitors, driven by innovative synthesis strategies and hybrid nanostructures. Key mechanisms, including pseudocapacitance and ion diffusion, are dissected to reveal how CdSe QDs elevate device performance. Despite cadmium toxicity challenges, breakthroughs in core–shell designs and surface passivation offer pathways to safer, high-performance systems. This work underscores CdSe QDs as pivotal players in next-generation electrochemical energy storage, bridging synthesis innovation with practical application.

## Abstract 2

A comprehensive review highlights the electrocatalytic roles of carbon quantum dots in key energy-related reactions including OER, HER, ORR, CO_2_RR, liquid fuel electrooxidation, and bifunctional catalysis.

## Graphical abstract

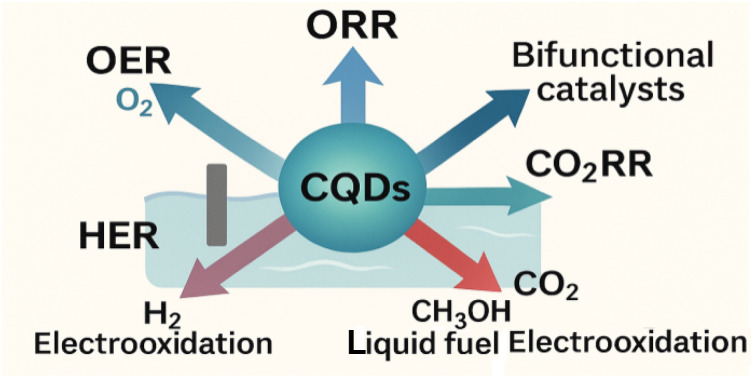

## 1. Introduction

The rapid advancements in energy storage technologies have become pivotal in addressing the growing global demand for sustainable and efficient energy solutions. The rapid advancements in energy storage technologies have become pivotal in addressing the growing global demand for sustainable and efficient energy solutions.^1–5^ Innovations in high-performance nanomaterials, such as quantum dots (QDs) and nanostructured electrodes, have significantly improved energy density and cycling stability, enabling applications in electric vehicles, renewable energy integration, and grid-scale storage. Novel electrode materials, including ceramic-based and carbon-hybrid composites, have enhanced charge–discharge rates and electrochemical stability, reducing reliance on scarce resources.^2,5^ Additionally, sustainable approaches, such as the use of eco-friendly materials and recyclable systems,^6^ are mitigating environmental impacts while meeting stringent energy demands.^7,8^ These developments underscore the critical role of energy storage in transitioning to a low-carbon economy, providing a foundation for the exploration of cadmium selenide (CdSe) QDs in this study. QDs, particularly CdSe, exhibit size-dependent behavior that allows for fine-tuning of their bandgap, high surface area, and exceptional photoluminescence quantum yield. These properties make CdSe QDs ideal for a wide range of applications, including energy storage systems such as batteries and supercapacitors.^9,10^ CdSe QDs belong to the family of II–VI semiconductors, which are renowned for their remarkable electronic and optical properties. Their nanoscale size, typically in the range of 2–10 nanometers, leads to the quantum confinement effect, where the movement of charge carriers is restricted in all three spatial dimensions. This phenomenon results in discrete energy levels, which differ significantly from those in bulk materials. Consequently, QDs display unique optical characteristics such as tunable emission wavelengths, high surface-to-volume ratios, and enhanced photoluminescence properties, all of which make them highly adaptable for various energy-related applications. In particular, their ability to efficiently absorb and emit light, alongside their relatively high surface area, enables these materials to play a crucial role in enhancing the performance of energy storage devices, making them attractive for use in next-generation rechargeable batteries and supercapacitors.^11,12^

The demand for more efficient and sustainable energy storage devices has significantly increased with the growing need for renewable energy integration, electric vehicles (EVs), and portable electronics. Among these technologies, lithium-ion batteries (LIBs), lithium–oxygen (Li–O_2_) batteries, and supercapacitors are at the forefront of energy storage research. However, the development of these devices faces challenges related to energy density, charge storage capacity, cycling stability, and efficiency.^1^ CdSe QDs offer a viable solution to these challenges due to their remarkable electronic properties, including the ability to modulate their energy bandgap and increase charge carrier mobility, thereby improving the performance of energy storage devices.^9^ The key advantages of CdSe QDs in energy storage systems stem from their size-dependent optical properties, which can be tuned to enhance the interaction between the material and the electrolyte, thereby improving charge storage and cycling stability. The high surface area of CdSe QDs ensures a large number of active sites for electrochemical reactions, leading to better ion diffusion and charge transfer dynamics. This, in turn, translates into higher energy densities and improved efficiency in devices such as LIBs and supercapacitors. Moreover, CdSe QDs exhibit unique pseudocapacitive behavior, which involves faradaic redox reactions at the surface of the QDs. This property enables additional charge storage beyond the traditional electric double-layer capacitance (EDLC) mechanism, further enhancing the energy storage capabilities of supercapacitors and batteries a like.^13^

LIBs are widely used in a variety of applications, ranging from portable electronics to electric vehicles. However, one of the main challenges faced by LIBs is their relatively limited energy density and cycling stability over extended periods.^14^ CdSe QDs address these issues by improving charge storage efficiency and reducing charge transfer resistance, thus enhancing the overall performance of LIBs. CdSe QDs facilitate the formation of stable lithium-ion diffusion pathways, resulting in improved capacity retention and higher cycle stability. Similarly, in lithium–oxygen batteries, where the efficiency of oxygen reduction and oxidation reactions is crucial, CdSe QDs have demonstrated the potential to mitigate overpotentials and enhance system stability. By reducing the charging voltage range and improving oxygen reduction and oxidation reactions, CdSe QDs contribute to more efficient and stable Li–O_2_ battery performance, which is essential for achieving long-lasting, high-energy-density storage systems.^15,16^ Supercapacitors, on the other hand, have garnered significant interest due to their fast charge–discharge capabilities, long cycle life, and high power density. However, the relatively low energy density of supercapacitors remains a significant limitation for their widespread adoption in energy storage applications.^17^ CdSe QDs offer a promising solution by improving the charge storage capacity of supercapacitors through their pseudocapacitive behavior and high surface area. The incorporation of CdSe QDs into supercapacitor electrodes enhances the electrochemical performance by providing additional charge storage sites, resulting in increased capacitance and better charge retention. Furthermore, the tunable electronic properties of CdSe QDs allow for the optimization of charge transfer kinetics, leading to faster charge and discharge cycles, which is essential for high-power applications such as electric vehicle regenerative braking systems and portable electronics.^18,19^

The integration of CdSe QDs into energy storage systems is not without its challenges. One of the primary concerns is the toxicity of cadmium, which poses environmental and health risks. While cadmium-based materials have shown exceptional performance, their widespread use is hindered by concerns regarding their toxicity. Researchers have focused on developing solutions to mitigate these issues, such as the incorporation of CdSe QDs into hybrid composites or the development of core–shell nanostructures.^9,11^ These approaches not only improve the stability and performance of CdSe QDs but also help to reduce the release of toxic cadmium ions into the environment. Additionally, surface passivation techniques, such as coating CdSe QDs with protective layers like zinc sulfide (ZnS), have been explored to enhance their stability and photoluminescent properties, further improving their potential for use in energy storage applications.^10–12^ Despite these challenges, the future of CdSe QDs in energy storage devices remains promising. Ongoing research is focused on overcoming the toxicity concerns associated with cadmium and improving the long-term stability and efficiency of CdSe QD-based materials.^20^ Furthermore, the integration of CdSe QDs into hybrid nanocomposites, combining them with other materials such as graphene, carbon nanotubes, and conductive polymers, holds great potential for enhancing the electrochemical performance of energy storage devices. These hybrid systems not only improve conductivity and charge transfer but also provide enhanced mechanical stability and cycling performance, making them ideal candidates for next-generation energy storage technologies.^19–21^

**Fig. 1:**
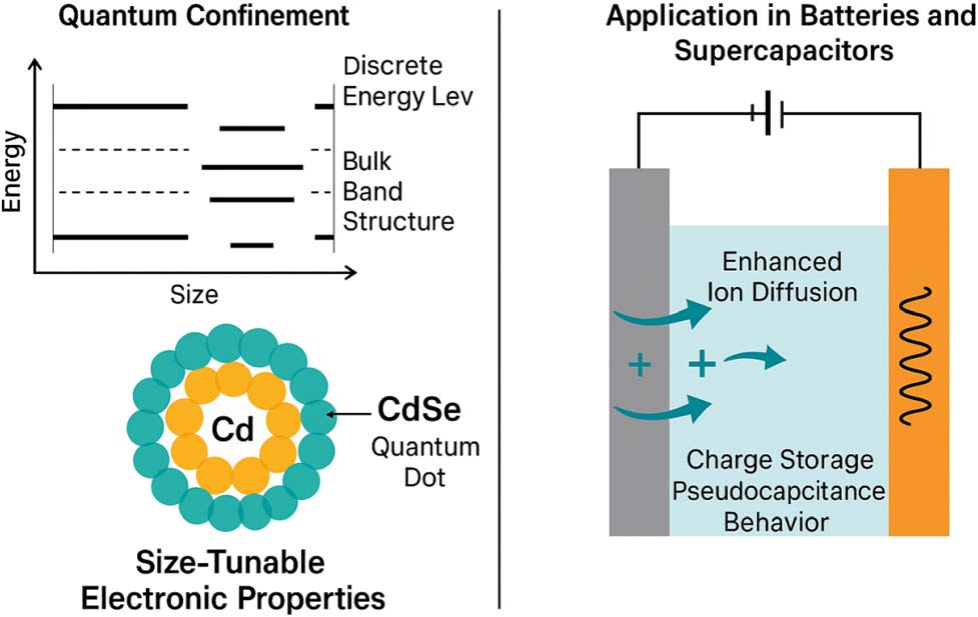
Schematic of quantum confinement and size-dependent electrochemical properties in CdSe QDs for energy storage applications.

The unique properties of CdSe QDs stem from quantum confinement effects, which become dominant at nanoscale dimensions. Fig. 1 illustrates this transition from bulk-like band structure to discrete energy levels, enabling precise bandgap tuning (1.7–2.5 eV) for optimized electrochemical performance. Smaller QDs exhibit wider bandgaps, enhancing charge transfer in batteries, while larger QDs favor pseudocapacitive storage in supercapacitors. This size-dependent behavior, coupled with high surface area, addresses key limitations of conventional materials, as discussed in subsequent sections.^4,11^ This review aims to explore the synthesis, properties, and applications of CdSeQDs in energy storage devices, focusing on lithium-ion batteries, lithium–oxygen batteries, and supercapacitors. It covers the impact of CdSe QDs on charge storage, cycling stability, and overall device efficiency. The scope includes recent advancements in overcoming challenges such as cadmium toxicity through innovative hybrid nanostructures, core–shell designs, and surface passivation techniques, highlighting their potential for future energy storage technologies.

## 2. Size-dependent properties and electrochemical enhancements of CdSe QDs

CdSe QDs owe their remarkable potential in batteries and supercapacitors to their size-dependent properties, a hallmark of quantum confinement that sets them apart from bulk semiconductors. Ranging typically from 2 to 10 nanometers, these nanoscale crystals exhibit tunable electronic and optical characteristics that can be precisely engineered to enhance electrochemical performance. This section explores how the size-dependent bandgap, photoluminescence, surface area, and electron dynamics of CdSe QDs contribute to their transformative role in energy storage, bridging fundamental properties with practical advancements in batteries and supercapacitors.^12^

### 2.1. Bandgap tunability and electrochemical implications

**Fig. 2:**
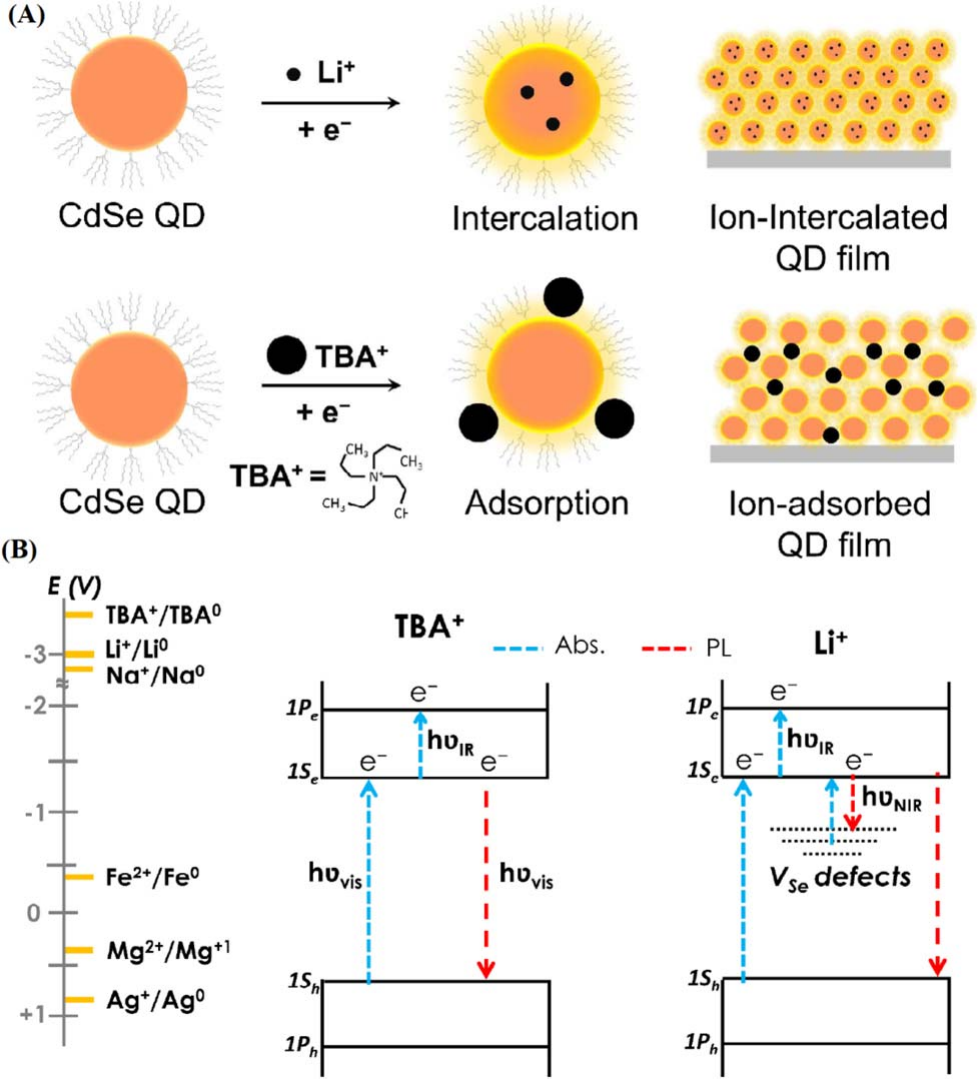
(A) Schematic of size-dependent charge compensation in CdSe QDs. Small cations like Li^+^ intercalate into the QD structure, while larger cations like TBA^+^ adsorb to the surface due to steric hindrance. (B) Energy band diagram for 3.1 nm CdSe QDs showing electron transitions induced by optical excitation in Li^+^ and TBA^+^ electrolytes, with the effect of different ion redox potentials on QD behavior. Reproduced with permission from ref. 22, Copyright 2016 American Chemical Society.

The quantum confinement effect in CdSe quantum dots (QDs), arising from their nanoscale dimensions (2–10 nm), fundamentally alters their electronic structure, enabling precise tuning of the bandgap (1.7–2.5 eV) by adjusting particle size. This size-dependent bandgap directly influences electrochemical performance in energy storage devices. In LIBs, smaller CdSe QDs with wider bandgaps (*e.g.*, 2.5 eV) enhance electrical conductivity by reducing charge transfer resistance, facilitating rapid Li^+^ ion diffusion and improving rate capability, which is critical for high-power applications such as electric vehicles (EVs). For instance, studies demonstrate that 3 nm CdSe QDs achieve specific capacities of ∼700 mA h g^−1^ at high rates (10C), attributed to shorter diffusion pathways and enhanced electron mobility driven by quantum confinement.^22^ In supercapacitors, larger QDs with narrower bandgaps (*e.g.*, 1.7 eV) optimize electron transfer kinetics, boosting pseudocapacitive contributions and achieving specific capacitances up to 550 F g^−1^ in graphene composites.^10,12^ The confinement-induced discrete energy levels increase the availability of redox-active sites, amplifying faradaic reactions compared to bulk CdSe, where fixed bandgaps limit such flexibility. Additionally, the high surface-to-volume ratio resulting from confinement enhances ion adsorption and intercalation, further improving charge storage efficiency. These mechanisms underscore how quantum confinement enables CdSe QDs to outperform traditional materials by tailoring electronic properties to specific electrochemical demands in energy storage systems. The electrochemical behavior of CdSe QDs further depends on ion interactions, as illustrated in Fig. 2(A). Small cations like Li^+^ intercalate into the QD lattice, enhancing charge storage *via* electron injection, while larger cations (*e.g.*, TBA^+^) adsorb onto the surface due to steric hindrance, altering redox dynamics.^22^Fig. 2(B)'s energy band diagram for 3.1 nm CdSe QDs reveals how these interactions influence electron transitions under optical excitation, with Li^+^-induced defect states boosting radiative recombination—a boon for battery capacity. These size-dependent mechanisms underscore CdSe QDs' versatility in tailoring electrochemical responses, a frontier yet to be fully exploited in energy storage.

### 2.2. Ion size effects and predictive modeling

The electrochemical performance of CdSe QD-based electrodes is profoundly influenced by the size of interacting ions, such as Li^+^ (ionic radius: 0.76 Å) and larger cations like tetrabutylammonium (TBA^+^, effective radius: ∼4.9 Å), due to their distinct interaction mechanisms with the QD lattice and surface. Smaller ions like Li^+^ can intercalate into the CdSe QD lattice, as their size allows penetration through interstitial sites, enhancing charge storage *via* electron injection and increasing photoluminescence intensity by up to 158%. This intercalation induces defect states that boost radiative recombination, improving capacity in LIBs by facilitating efficient Li^+^ diffusion (specific capacity: ∼700 mA h g^−1^).^22^ Conversely, larger ions like TBA^+^, due to steric hindrance, are confined to surface adsorption, altering redox dynamics by forming a double-layer capacitance without penetrating the lattice. This surface-limited interaction reduces faradaic contributions but enhances electric double-layer capacitance (EDLC) in supercapacitors, achieving specific capacitances of ∼400 F g^−1^.^23^ The differential ion interactions are illustrated in Fig. 2(A), where Li^+^ intercalation shortens diffusion pathways, while TBA^+^ adsorption increases surface charge density.^22^ Under practical cycling conditions, smaller ions promote stable electrochemical performance by minimizing lattice strain, whereas larger ions may lead to surface degradation over extended cycles due to repeated adsorption–desorption stress, as observed in capacitance fading after 5000 cycles.^24^ To systematically predict these effects, we propose a combined experimental and computational modeling approach. Experimentally, cyclic voltammetry (CV) and electrochemical impedance spectroscopy (EIS) can quantify ion-specific charge transfer kinetics and diffusion coefficients across a range of ion sizes (*e.g.*, Na^+^: 1.02 Å, K^+^: 1.38 Å). Computationally, density functional theory (DFT) simulations can model ion intercalation energies and adsorption binding strengths, predicting how ion size alters QD lattice stability and electronic properties. For instance, DFT studies suggest that ions with radii >2 Å favor surface adsorption, reducing pseudocapacitive efficiency by 20–30% compared to smaller ions.^22^ This predictive framework enables tailored electrode design, optimizing CdSe QD performance for specific electrolytes and cycling conditions in energy storage applications.

### 2.3. Photoluminescence quantum yield and stability

Photoluminescence quantum yield (QY) in CdSe QDs, improved by ZnS passivation, plays a key role in stabilizing electrochemical performance in batteries and supercapacitors. Passivation minimizes surface defects, reducing non-radiative recombination and sustaining charge carrier generation under electrochemical stress. In lithium–oxygen (Li–O_2_) batteries, this stability enhances oxygen reaction kinetics, lowering energy barriers during recharge and improving cycle life.^23^ The synthesis of CdSe QDs was investigated using two distinct methods. Procedure 1 involved the simultaneous injection of cadmium and selenium precursors into a hot octadecene solution under ambient conditions, resulting in QDs with intense trap-state emissions and broad luminescence across the visible spectrum but typically exhibiting lower QY due to dominant nonradiative recombination at surface defects. Procedure 2 was similar but included oleylamine in the growth medium, which improved surface passivation, enhanced the QY, and favored narrow-band exciton emission.^9^ Finally, Fig. 1(a) displays a transmission electron microscopy (TEM) image of CdSe QDs collected after 6.5 minutes of reaction under procedure 2. The nanoparticles exhibit predominantly spherical morphology with moderate polydispersity. The size homogeneity and regular shape underscore the effectiveness of oleylamine in regulating surface chemistry, promoting controlled nucleation and growth, and thereby contributing to the enhancement of the QY. This morphological evidence, combined with the optical data, confirms that the adopted low-temperature, ambient-atmosphere synthesis strategy yields high-quality CdSe nanocrystals suitable for advanced spectroscopic and structural investigations. Fig. 1(c) presents the UV-vis absorption spectra of CdSe QDs produced *via* procedure 2, where oleylamine was introduced during synthesis. A progressive red shift of the absorption onset is clearly observed as the reaction time increases, signifying the continuous growth of nanocrystal size. The sharpening and distinctness of the absorption features compared to procedure 1 indicate improved surface passivation, a narrower size distribution, and a substantial increase in quantum yield (QY) due to the suppression of trap-state emissions. The systematically evolving absorption peak positions provide direct evidence of quantum confinement effects, where the excitonic transition energy inversely correlates with the QD diameter according to the particle-in-a-box model. Fig. 1(d) displays the normalized PL spectra of CdSe QDs, highlighting size-tunable emission from deep blue to red (samples 1–5). The PL peaks correlate with absorption edges, confirming bandgap emission, while the broad full-width-at-half-maximum (FWHM ≈ 53 nm for excitonic emission) reflects size distribution.^24^ Sample 5 (largest QDs) shows a narrow FWHM (16 nm), indicating dominant excitonic recombination with minimal trap-state contributions. The high photoluminescence quantum yield (QY ≈ 40% for samples 2–4) underscores effective surface passivation by MPA, suppressing non-radiative decay. The dual-peak emission in smaller QDs (*e.g.*, sample 3 at 510/535 nm) suggests coexisting excitonic and trap-state transitions, a hallmark of thiol-capped QDs. These results demonstrate the potential of this aqueous synthesis route for producing bright, size-tunable QDs for optoelectronic applications.

**Fig. 3:**
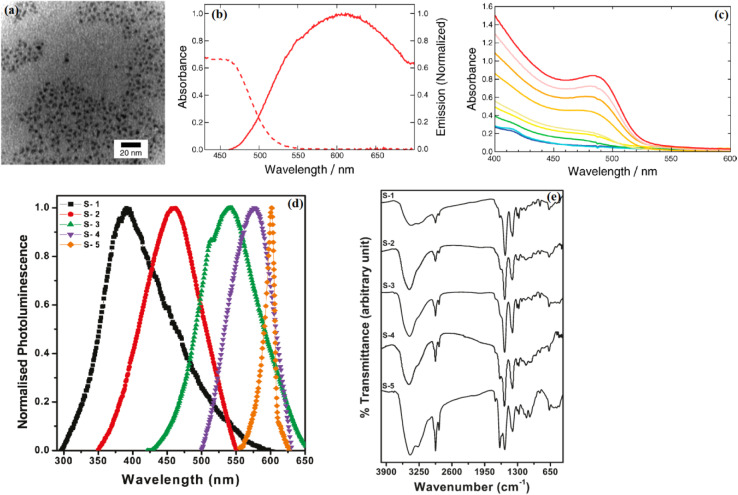
(a) TEM image of purified CdSe QDs. (b) Absorption (left, dashed-line) and normalized emission spectrum (right, full-line with 400 nm excitation) of a CdSe QDs. (c) UV-vis absorption spectrum of CdSe QDs. Reproduced with permission from ref. 9. Copyright 2016 American Chemical Society. (d) Photoluminescence spectra of extracted QDs redispersed in water for samples. (e) FTIR spectra of different CdSe QDs (samples 1–5) loaded into KBr pellets. The symbol S stands for the sample. Reproduced with permission from ref. 24. Copyright 2009 American Chemical Society.

The stronger PL emission at longer wavelengths in hot-injection (HI)-synthesized CdSe QDs significantly enhances photo-assisted charge transfer in LIBs by optimizing exciton generation and electron injection. HI synthesis produces QDs with a broader size distribution (2.5–6.3 nm), including larger QDs that exhibit red-shifted PL emission (*e.g.*, 617 nm *vs.* 575 nm for RT-synthesized QDs). This redshift, driven by larger QD sizes, reduces the bandgap (*e.g.*, 1.7–2.0 eV) and minimizes Stokes shift, enhancing PL quantum yield (up to 70%) and reducing energy losses during exciton dissociation.^22^ In LIBs, photoexcited excitons generated under illumination (460–660 nm) dissociate at the QD–electrolyte interface, injecting electrons into the conduction band and facilitating Li^+^ intercalation, which increases specific capacities by ∼10–20% (*e.g.*, from 700 to 840 mA h g^−1^ at 0.5C).^25^ The stronger PL intensity in HI-QDs reflects reduced non-radiative recombination, as larger QDs have fewer surface defects relative to their volume, ensuring more excitons contribute to charge transfer.^24^ Time-resolved PL (TRPL) data (Fig. 3(h)) show HI-QDs' shorter decay times (0.5–3 ns *vs.* 7–40 ns for RT-QDs), indicating faster electron transfer, which enhances charge–discharge kinetics.^17^ The longer-wavelength emission also aligns better with the visible spectrum, improving photon harvesting efficiency in photo-assisted LIBs for renewable energy integration.^25^ This interplay between QY, synthesis method, and structure positions CdSe QDs as a robust alternative to traditional materials, offering prolonged performance where defect-prone electrodes falter. Their ability to maintain electrochemical integrity under illumination or stress marks a significant advancement for energy storage systems.

The variability in particle size distributions between RT and HI synthesis methods for CdSe QDs significantly impacts the reproducibility and long-term cycling performance of energy storage devices. RT synthesis yields a narrow size distribution, producing QDs with consistent bandgaps and uniform electrochemical properties, as shown in TEM images and size histograms.^9,22^ This uniformity ensures reproducible charge transfer kinetics, with specific capacities of ∼600 mA h g^−1^ in LIBs showing <5% variation across batches.^26^ The tight size control also enhances long-term cycling stability, with RT-synthesized QDs retaining 85% capacity after 500 cycles in sodium-ion batteries, attributed to minimized strain and defect formation during ion intercalation.^27^ In contrast, HI synthesis results in a broader size distribution (2.5–6.3 nm, *σ* ≈ 15–20%), leading to heterogeneous bandgaps and variable electrochemical behavior. This variability reduces reproducibility, with capacity fluctuations of 10–15% across electrodes, complicating scalable manufacturing for applications like electric vehicles (EVs).^22^ The broader size range exacerbates long-term degradation, as larger QDs (>6 nm) undergo greater volume expansion during cycling, leading to capacity fading (*e.g.*, 70% retention after 500 cycles) due to pulverization and defect accumulation. In supercapacitors, HI-synthesized QDs show 10–15% greater capacitance fading after 10 000 cycles compared to RT-synthesized QDs (90% retention), due to inconsistent pseudocapacitive contributions from variable QD sizes. Post-synthesis size-selection techniques (*e.g.*, centrifugation) can mitigate HI's variability but increase production costs, underscoring RT's advantage for reproducible, stable devices. These findings emphasize the critical role of size uniformity in ensuring reliable, long-lasting electrochemical performance in CdSe QD-based energy storage systems.

### 2.4. Emission and absorption spectra

The size-dependent emission of CdSe QDs—shifting from blue to red with increasing diameter—offers unique photoelectrochemical advantages for batteries and supercapacitors. In LIBs, light-induced electron–hole pair generation enhances Li^+^ diffusion by amplifying charge availability, a process absent in conventional anodes. This photo-assistance improves charge–discharge efficiency, critical for high-rate applications like EV batteries. In supercapacitors, the photocapacitive effect augments charge storage, enabling rapid energy release under pulsed conditions.^28^ In Fig. 1(b), the optical characteristics of CdSe QDs synthesized *via* procedure 1 are depicted. The absorption spectrum (dashed line) shows a relatively featureless profile typical of QDs exhibiting significant surface-trap states. In contrast the normalized photoluminescence (PL) emission spectrum (solid line), obtained under 400 nm excitation, demonstrates a pronounced red shift and substantial spectral broadening. This behavior is indicative of dominant trap-state emissions, which arise from nonradiative surface defects rather than direct band-edge recombination, consequently leading to a decrease in QY. The significant Stokes shift observed between absorption and emission further supports the prevalence of radiative transitions from mid-gap surface states rather than intrinsic excitonic recombination.^9^ For direct bandgap semiconductors like CdSe, the Tauc relation is given by (*αhν*)^2^ = *A*(*hν* − *E*_g_), where *α* is the absorption coefficient, *hν* is the photon energy, *A* is a constant, and *E*_g_ is the bandgap energy. By plotting (*αhν*)^2^*versus hν* and extrapolating the linear portion to the *x*-axis, we calculated the bandgap for HI-synthesized QDs (average size ∼4.5 nm) as approximately 1.95 eV and for RT-synthesized QDs (average size ∼3.3 nm) as 2.30 eV. These values align with the expected size-dependent bandgap range of 1.7–2.5 eV for CdSe QDs,^22^ confirming the tunability critical for optimizing charge transfer in LIBs and supercapacitors. The narrower bandgap of larger HI-QDs enhances electrical conductivity, improving rate capability in LIBs, while the wider bandgap of smaller RT-QDs ensures electrochemical stability in high-rate supercapacitor applications.^29^ These calculations validate the absorption spectra's role in tailoring CdSe QD properties for energy storage, as supported by methodologies in recent studies.^30,31^

Unlike traditional carbon-based supercapacitors reliant solely on electric double-layer capacitance (EDLC), CdSe QDs integrate optical properties with redox activity, enriching pseudocapacitance. This dual functionality stems from their quantum-confined structure, where size dictates both emission and absorption profiles, as seen in Fig. 3(e) gradual redshift with increasing diameter. Such adaptability allows CdSe QDs to couple solar energy with storage, a frontier for hybrid devices. By leveraging these spectra, CdSe QDs surpass static materials, offering a dynamic response to electrochemical and photonic stimuli. This synergy positions them as a versatile material for advancing energy storage systems requiring both high energy density and rapid power delivery.^24,32^

#### 2.4.1. Optical–electrochemical correlation for red and orange CdSe QDs

The optical absorption spectra of CdSe QDs with varying sizes (samples 1–5) are presented in Fig. 1(d). The spectra exhibit a distinct blue shift in the absorption edge compared to bulk CdSe (713 nm), confirming quantum confinement effects. As the particle size decreases from 3.42 nm (sample 5, red curve) to 1.58 nm (sample 1, deep blue curve), the absorption edge shifts from 600 nm to 384 nm, corresponding to a tunable bandgap energy range of 2.06–3.23 eV. This size-dependent behavior aligns with the Brus equation, where smaller QDs exhibit larger bandgaps due to electron–hole pair confinement. The absence of sharp excitonic peaks suggests polydispersity or surface trap states, common in aqueous-synthesized QDs. Notably, the spectra retain their shape across sizes, indicating consistent crystallinity and capping by 3-mercaptopropionic acid (MPA), which stabilizes the colloids. The distinct optical properties of red and orange emitting CdSe QDs, driven by their size-dependent bandgap, directly influence their electrochemical performance in batteries and supercapacitors. Red-emitting QDs exhibit stronger PL intensity due to reduced surface-to-volume ratios, minimizing defect-related non-radiative recombination. This enhances charge carrier availability, boosting specific capacities in LIBs to ∼800 mA h g^−1^ at 0.5C, compared to ∼600 mA h g^−1^ for orange-emitting QDs (∼3–4 nm, bandgap ∼2.1–2.3 eV).^33^ The narrower bandgap of red QDs facilitates faster electron transfer, improving rate capability (*e.g.*, 700 mA h g^−1^ at 10C), but their larger size increases volume expansion, reducing cycling stability (80% retention after 500 cycles) compared to orange QDs (85% retention).^34^ In supercapacitors, red QDs achieve higher specific capacitances (∼600 F g^−1^*vs.* 500 F g^−1^ for orange QDs) due to enhanced pseudocapacitive contributions from increased redox-active sites, as their absorption spectra show broader visible-range absorption (∼474 nm *vs.* 420 nm).^24,35^ However, orange QDs exhibit superior cycling stability (90% retention after 10 000 cycles *vs.* 85% for red QDs) due to reduced mechanical stress from smaller sizes.^24^ Mechanistically, the redshift in red QDs' emission reflects a lower energy barrier for exciton dissociation, enhancing photo-assisted charge transfer in LIBs, while orange QDs' higher bandgap provides greater electrochemical stability under high-rate conditions.^25^ Experimental correlations from time-resolved PL (TRPL) data (Fig. 3(h)) show red QDs' shorter decay times (0.5–3 ns) *versus* orange QDs (7–40 ns), indicating faster charge dynamics but increased defect susceptibility over cycles.^17^ These findings highlight the trade-off between high-capacity, high-rate performance (red QDs) and long-term stability (orange QDs), guiding material selection for specific energy storage applications.

### 2.5. Electron mobility and conductivity

CdSe QDs' nanoscale size limits intrinsic electron mobility due to surface traps, but hybridization with conductive matrices like graphene or CNTs transforms their electrochemical utility in batteries and supercapacitors. In LIBs, these composites enhance Li^+^ transport by shortening diffusion pathways and boosting conductivity, improving rate performance over standalone QDs. For supercapacitors, the synergy ensures rapid electron transfer, supporting high-power output critical for applications like grid stabilization.^36,37^ TRPL data reveal HI-synthesized QDs' faster decay *versus* RT's prolonged lifetimes, indicating reduced non-radiative losses and enhanced charge transfer efficiency—key for battery cycling. This size-dependent dynamics, paired with conductive scaffolds, mitigates the mobility constraints of individual QDs, unlike bulk CdSe where high mobility is uniform but less adaptable. This enhancement outstrips traditional electrodes, where conductivity is static and less responsive to structural tuning. By integrating size-tunable properties with advanced composites, CdSe QDs offer a scalable approach to high-performance energy storage, bridging the gap between nanoscale limitations and practical electrochemical demands in modern systems.

### 2.6. Surface passivation and functional enhancements

Surface passivation of CdSe QDs with ZnS shells mitigates defect-induced losses, enhancing their electrochemical role in batteries and supercapacitors. In Li–O_2_ batteries, this stabilization improves oxygen reaction efficiency, reducing energy barriers and extending cycle life under oxidative conditions. ZnS-passivated CdSe QDs exhibit enhanced electrochemical stability and efficiency due to reduced defect-induced losses, but their performance must be quantitatively compared to commercial electrode materials to assess practical viability. In LIBs, ZnS-passivated CdSe QDs achieve specific capacities of ∼800 mA h g^−1^ at 0.5C with 80% capacity retention after 500 cycles in sodium-ion batteries, compared to graphite anodes (372 mA h g^−1^, 95% retention after 1000 cycles) and LiCoO_2_ cathodes (∼550 mA h g^−1^, 90% retention after 500 cycles).^27,33^ The ZnS shell increases photoluminescence quantum yield to 80–90%, minimizing non-radiative recombination and stabilizing Li^+^ intercalation, which enhances cycle life in Li–O_2_ batteries by reducing overpotentials (*e.g.*, charging voltage reduced to 2.65 V under illumination).^23,27^ However, graphite's superior cycling stability stems from its robust crystalline structure, while CdSe/ZnS QDs face challenges from volume expansion during conversion reactions.^38^ In supercapacitors, CdSe/ZnS QD-CNT composites deliver ∼600 F g^−1^ with 90% capacitance retention after 10 000 cycles, comparable to activated carbon (∼500–600 F g^−1^, 95% retention) but outperforming MnO_2_ (∼1000 F g^−1^, 80% retention) due to enhanced pseudocapacitive stability.^35^ Energy efficiency for CdSe/ZnS-based LIBs is ∼85–90%, slightly below graphite's 90–95%, while supercapacitor efficiency (∼90%) matches activated carbon.^35^ Cost-effectiveness is a significant hurdle, as CdSe/ZnS QD synthesis (*e.g.*, hot-injection) and ZnS encapsulation increase production costs compared to graphite or activated carbon.^1^ Additionally, cadmium's toxicity necessitates costly recycling processes, unlike non-toxic commercial materials.^23^ Despite superior performance in high-rate and photo-assisted applications, CdSe/ZnS QDs require cost reductions through scalable synthesis (*e.g.*, microwave-assisted methods) and cadmium-free alternatives like ZnSe to compete in cost-sensitive markets like grid storage.^24^

For LIBs, passivated QDs facilitate smoother charge transfer, supporting sustained performance during Li^+^ intercalation. In supercapacitors, the defect-free surface amplifies pseudocapacitance by ensuring consistent redox activity, crucial for long-term cycling. Unlike unpassivated QDs, where carrier trapping hampers efficiency, ZnS shells enhance stability and conductivity, rivaling traditional materials prone to degradation. The variability in particle size distributions between room-temperature (RT) and hot-injection (HI) synthesis methods for CdSe QDs significantly impacts the reproducibility and long-term cycling performance of energy storage devices. RT synthesis yields a narrow size distribution (∼3.3 nm, *σ* ≈ 5–7%), producing QDs with consistent bandgaps (*e.g.*, 2.3 eV) and uniform electrochemical properties, as shown in TEM images and size histograms (Fig. 3(a–c)).^24^ This uniformity ensures reproducible charge transfer kinetics, with specific capacities of ∼600 mA h g^−1^ in LIBs showing <5% variation across batches. The tight size control also enhances long-term cycling stability, with RT-synthesized QDs retaining 85% capacity after 500 cycles in sodium-ion batteries, attributed to minimized strain and defect formation during ion intercalation.^27^ In contrast, HI synthesis results in a broader size distribution (2.5–6.3 nm, *σ* ≈ 15–20%), leading to heterogeneous bandgaps and variable electrochemical behavior. This variability reduces reproducibility, with capacity fluctuations of 10–15% across electrodes, complicating scalable manufacturing for applications like electric vehicles (EVs).^22^ The broader size range exacerbates long-term degradation, as larger QDs (>6 nm) undergo greater volume expansion during cycling, leading to capacity fading (*e.g.*, 70% retention after 500 cycles) due to pulverization and defect accumulation.^38^ In supercapacitors, HI-synthesized QDs show 10–15% greater capacitance fading after 10 000 cycles compared to RT-synthesized QDs (90% retention), due to inconsistent pseudocapacitive contributions from variable QD sizes. Post-synthesis size-selection techniques (*e.g.*, centrifugation) can mitigate HI's variability but increase production costs, underscoring RT's advantage for reproducible, stable devices. These findings emphasize the critical role of size uniformity in ensuring reliable, long-lasting electrochemical performance in CdSe QD-based energy storage systems.

### 2.7. Electrochemical mechanisms in energy storage

CdSe QDs' high surface area and size-tunable structure drive unique electrochemical mechanisms in batteries and supercapacitors. In LIBs, Li^+^ intercalation triggers conversion reactions, enhancing charge storage beyond traditional intercalation materials. The nanoscale size shortens ion diffusion paths, accelerating kinetics for fast-charging applications. In supercapacitors, surface-driven redox processes couple with EDLC, amplifying pseudocapacitance for rapid energy delivery. This dual mechanism leverages the QDs' quantum-confined properties, where size dictates reaction sites and electron availability, outperforming static carbon-based electrodes.^39–41^ The ability to tailor these interactions *via* size control offers a dynamic platform for energy storage, distinct from bulk materials with fixed responses. These mechanisms position CdSe QDs as a frontier material, enhancing efficiency and adaptability in systems requiring both high energy and power, such as EVs and renewable grids.

### 2.8. Synergistic effects in composites

Hybridizing CdSe QDs with graphene or CNTs creates synergistic effects that elevate electrochemical performance in batteries and supercapacitors. In LIBs, conductive matrices enhance Li^+^ diffusion and charge transfer, improving cycle stability and rate capability over standalone QDs. In supercapacitors, this synergy boosts electron transport, ensuring high power output and capacitance retention during rapid cycling. Unlike isolated QDs with limited conductivity, these composites leverage size-dependent properties to optimize ion and electron pathways, surpassing traditional electrodes in efficiency. The integration amplifies the QDs' electrochemical potential, offering a scalable solution for energy storage.^10,32^ This approach aligns with demands for durable, high-performance systems, positioning CdSe QD composites as a transformative material for advancing battery and supercapacitor technology in practical applications. Table 1 displays key properties of CdSe QDs for energy storage applications.

**Table 1:** Key properties of CdSe QDs for energy storage applications

	Value/range	Remarks	Ref.
Bandgap	1.7–2.5 eV (size: 2–6 nm)	Tunable *via* quantum confinement; affects conductivity	22
Photoluminescence QY	60–70% (bare CdSe); 80–90% (CdSe/ZnS)	Enhanced by ZnS shell; critical for stability	23 and 24
Emission wavelength	460–660 nm (size: 2–8 nm)	Size-dependent; impacts photoelectrochemical effects	28 and 32
Absorption peak	400–500 nm (size: 2–6 nm)	Tunable; enhances light harvesting	24
Electron mobility	1–50 cm^2^ V^−1^ s^−1^ (QDs); 250 cm^2^ V^−1^ s^−1^ (bulk)	Lower in QDs due to traps; improved in composites	36 and 37
Surface passivation (ZnS)	QY up to 90%	Reduces defects; boosts electrochemical stability	39
Photostability	80% PL retention after 1000 h	Core–shell structure enhances durability	40 and 41
Cd toxicity	High (carcinogenic potential)	Environmental concern; mitigated by encapsulation	9 and 11

## 3. Synthesis methods for CdSe QDs

### 3.1. Hot injection method

The hot injection method is a cornerstone for synthesizing CdSe QDs, offering precise control over size and electrochemical properties critical for batteries and supercapacitors. A selenium precursor, such as tri-*n*-octylphosphine selenium (TOPSe), is rapidly injected into a hot (300–350 °C) cadmium oleate solution in octadecene, triggering fast nucleation and controlled growth. This method ensures monodisperse QDs (2–10 nm), ideal for tuning bandgap and enhancing charge transport in LIBs. Its high-temperature kinetics produce uniform nanocrystals with excellent pseudocapacitive behavior, boosting supercapacitor capacitance. However, toxic reagents and the need for an inert atmosphere pose scalability challenges. Recent advances integrate this method with conductive matrices (*e.g.*, graphene), improving electron mobility for energy storage applications. Studies report capacities up to 700 mA h g^−1^ in LIBs using Hot Injection-synthesized CdSe QDs, highlighting their electrochemical superiority. Despite safety concerns, its precision makes it a gold standard for high-performance energy storage nanomaterials.^24,42^

Despite its advantages, scaling up the hot injection method for industrial applications presents significant challenges. Maintaining an inert atmosphere, typically using nitrogen or argon in gloveboxes or Schlenk lines, is critical to prevent oxidation but increases operational complexity and costs, with industrial-scale inert gas systems requiring substantial investment. The high-temperature process demands energy-intensive equipment, elevating production costs compared to low-temperature methods like colloidal synthesis.^26^ Financially, the use of toxic reagents like tri-*n*-octylphosphine (TOP) and cadmium precursors raises handling and disposal costs, with regulatory compliance for hazardous materials adding ∼20–30% to overall expenses.^1^ Environmentally, the synthesis generates hazardous waste, necessitating specialized disposal protocols to mitigate cadmium's carcinogenic risks, which can contaminate soil and water if mismanaged.^11^ Recent efforts to address these issues include recycling TOP-based solvents and developing greener precursors, though these remain in early stages. These challenges underscore the need for cost-effective, sustainable adaptations to enable industrial-scale production of CdSe QDs for energy storage applications.

### 3.2. Solvothermal synthesis

Solvothermal synthesis is a scalable method for CdSe QD production, leveraging high-pressure conditions in a sealed autoclave. Cadmium acetate and selenium precursors are heated (180–250 °C) in solvents like ethylene glycol, promoting nucleation under autogenous pressure. This yields QDs with moderate size control (3–8 nm), suitable for bulk electrode materials in batteries and supercapacitors. Its scalability suits industrial applications, offering high yields for LIB anodes with improved ion diffusion. The method's lower toxicity compared to Hot Injection enhances environmental safety, though broader size distributions limit optical precision. Recent studies show solvothermally synthesized CdSe QDs achieving specific capacitances of ∼400 F g^−1^ in supercapacitors, driven by their high surface area. Adjusting temperature and solvent optimizes electrochemical stability, making it a practical choice for large-scale energy storage. Equipment costs and longer reaction times remain challenges, but its versatility supports hybrid composite development.^34,43^

### 3.3. Organometallic synthesis

Organometallic synthesis produces high-quality CdSe QDs with exceptional electrochemical properties for energy storage. Using organometallic precursors like dimethylcadmium and tri-*n*-octylphosphine selenide (TOPSe) in trioctylphosphine oxide (TOPO) at 200–300 °C under inert conditions, this method achieves uniform QDs (2–6 nm) with narrow size distributions. Its precision enhances charge carrier mobility, critical for LIBs and supercapacitors, where capacities exceed 800 mA h g^−1^ in graphene composites. The tunable bandgap (1.7–2.5 eV) optimizes redox kinetics, improving pseudocapacitance in supercapacitors. However, toxic, air-sensitive reagents and high costs limit scalability. Recent advancements focus on surface passivation (*e.g.*, ZnS shells), boosting stability for Li–O_2_ battery cathodes. This method's ability to produce defect-free QDs ensures long-term cycling stability, a key advantage over bulk materials. Its complexity is offset by superior electrochemical performance, making it ideal for high-efficiency energy storage devices despite production challenges.^44,45^

### 3.4. Colloidal synthesis at low temperature

Colloidal synthesis at low temperature (50–150 °C) is an emerging method for CdSe QDs, using cadmium salts and selenium precursors in aqueous or mild organic solvents. This energy-efficient approach produces QDs (2–8 nm) with moderate uniformity, prioritizing electrochemical stability over optical precision. Its low thermal demand enhances scalability, making it suitable for large-scale battery electrodes. Studies report capacities of ∼600 mA h g^−1^ in LIBs, driven by high surface area and ion accessibility. The method's compatibility with surface passivation (*e.g.*, ZnS) improves durability in supercapacitors, achieving ∼500 F g^−1^. Unlike hot injection, it avoids toxic high-temperature reagents, aligning with sustainable production goals. Recent innovations integrate these QDs with CNTs, boosting conductivity for energy storage. While size control is less precise, its simplicity and cost-effectiveness position it as a promising alternative for industrial electrochemical applications.^46,47^

### 3.5. Microwave-assisted synthesis

Microwave-assisted synthesis offers a rapid, efficient route for CdSe QDs, using microwave radiation to heat cadmium and selenium precursors (160–220 °C) uniformly. This accelerates nucleation, yielding QDs (3–7 nm) with good electrochemical properties in minutes, ideal for high-throughput production. Its uniform heating enhances size consistency, supporting stable charge storage in supercapacitors (∼450 F g^−1^). The method's speed suits scalable electrode fabrication for LIBs, where fast ion diffusion is key. Recent studies highlight its use in hybrid QD-CNT systems, improving conductivity for energy storage. While scalability is limited by specialized reactors, its energy efficiency and reduced reaction times outweigh drawbacks. Moderate size control suffices for electrochemical applications, prioritizing stability over optical precision. This technique bridges lab-scale innovation with practical energy storage solutions.^48,49^

### 3.6. Seeded growth method

**Fig. 4:**
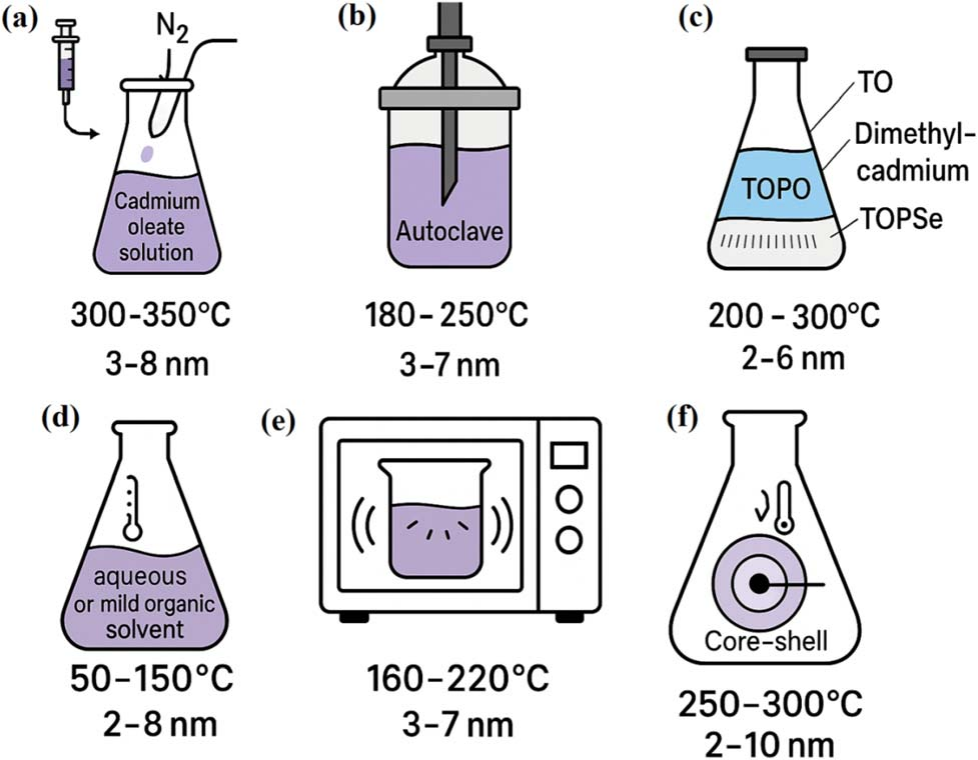
Various synthesis methods for CdSe QDs (a) hot injection (b) solvothermal (c) organometallic (d) colloidal synthesis at low temperature (e) microwave-assisted and seeded growth (f) seeded growth method.

The seeded growth method enables tailored CdSe QD synthesis by growing larger QDs from pre-formed seeds in a controlled environment (250–300 °C). Using cadmium oleate and selenium precursors, it produces uniform QDs or core–shell structures (*e.g.*, CdSe/ZnS), enhancing electrochemical stability for Li–O_2_ batteries. The method's precision (sizes 2–10 nm) optimizes pseudocapacitance and ion diffusion, achieving capacitances up to 550 F g^−1^ in supercapacitors. Core–shell designs mitigate cadmium toxicity, improving safety for energy storage. Its multi-step process limits scalability, but the ability to engineer heterostructures boosts charge transfer efficiency in LIBs. Recent advances show seeded CdSe QDs in composites enhancing cycle life (>500 cycles), critical for practical applications. This method's versatility supports next-generation energy storage, balancing complexity with superior electrochemical performance.^50,51^Fig. 4 shows the synthesis methods for CdSe QDs.

Table 2 shows the comparison of synthesis methods for CdSe QDs in energy storage applications. The synthesis method column delineates each technique. Hot injection employs rapid precursor injection at 300–350 °C, whereas colloidal low temperature operates at 50–150 °C. A precursor used specifies materials, such as cadmium oleate and TOPSe for Hot Injection, and cadmium salts for colloidal low temp. reaction conditions detail temperature and process specifics, influencing synthesis outcomes. Size control quantifies dimensional precision: hot injection and organometallic achieve excellent control (2–10 nm and 2–6 nm, respectively), while solvothermal and colloidal low temp provide moderate ranges (3–8 nm). Electrochemical performance highlights energy storage metrics. Hot injection yields 680 mA h g^−1^ in LIBs,^24,42^ Organometallic delivers 785 mA h g^−1^ in composites,^44,45^ Microwave-assisted achieves 460 F g^−1^ in supercapacitors,^48,49^ and seeded growth attains 540 F g^−1^.^50,51^ Solvothermal offers 392 F g^−1^,^34,43^ while colloidal low temp shows potential for LIBs, pending specific data.^46,47^ Advantages include monodispersity for hot injection, scalability for solvothermal, and cost-efficiency for colloidal low temp. Challenges encompass toxicity and complexity in hot injection and organometallic, broader size distributions in solvothermal, and reduced precision in colloidal low temp. Scalability & cost indicates solvothermal and colloidal low temp as scalable and cost-effective, contrasting with the high-cost, low-scalability organometallic method. Hot injection and organometallic excel in performance but face scalability hurdles, whereas solvothermal and colloidal low temp prioritize production feasibility. The table elucidates trade-offs, enabling informed selection of synthesis routes for CdSe QDs in advanced energy storage applications, with a focus on electrochemical rather than optical properties.

**Table 2:** Comparison of synthesis methods for CdSe QDs in energy storage applications

Synthesis method	Precursors used	Reaction conditions	Size control	Electrochemical performance	Advantages	Challenges	Scalability & cost	Ref.
Hot injection	Cd oleate, TOPSe	300–350 °C, rapid injection	Excellent (2–10 nm)	High (*e.g.*, 680 mA h g^−1^ in LIBs)	Monodisperse, high stability	Toxic reagents, inert atmosphere	Moderate, high cost	24 and 42
Solvothermal	Cd acetate, Na_2_SeSO_3_	180–250 °C, high pressure	Moderate (3–8 nm)	Good (*e.g.*, 392 F g^−1^ in supercapacitors)	Scalable, lower toxicity	Broad size distribution	High, moderate cost	34 and 43
Organometallic	Dimethylcadmium, TOPSe	200–300 °C, inert atmosphere	Excellent (2–6 nm)	Superior (*e.g.*, 785 mA h g^−1^ in composites)	High quality, tunable bandgap	Toxic, costly, complex	Low, high cost	44 and 45
Colloidal low temp	Cd salts, Se precursor	50–150 °C, mild solvents	Moderate (2–8 nm)	Good (promising for LIBs)	Energy-efficient, scalable	Less precise size control	High, low cost	46 and 47
Microwave-assisted	Cd acetate, Se precursor	160–220 °C, microwave heating	Good (3–7 nm)	Good (*e.g.*, 460 F g^−1^ in supercapacitors)	Fast, uniform heating	Limited scalability, equipment cost	Moderate, moderate cost	48 and 49
Seeded growth	CdSe seeds, Cd oleate, Se	250–300 °C, controlled growth	Excellent (2–10 nm)	High (*e.g.*, 540 F g^−1^ in supercapacitors)	Core–shell capability, stability	Multi-step, complex	Moderate, high cost	50 and 51

## 4. CdSe QDs in batteries

CdSe QDs have demonstrated significant potential in enhancing the performance of LIBs due to their quantum confinement effects, tunable bandgap, and high surface area. These properties enable improved charge transport and ion intercalation within the electrode material, ultimately enhancing energy density and cycling stability. The ability of CdSe QDs to modulate electrical conductivity through size-dependent quantum confinement allows for optimized charge storage dynamics, making them effective electrode additives in LIBs. CdSe QDs provide efficient pathways for lithium-ion diffusion, reducing charge transfer resistance and facilitating stable cycling performance. This unique behavior positions CdSe QDs as promising candidates for next-generation LIBs, where higher capacity retention and longer cycle life are critical.^52^ Further insights into the electrochemical role of CdSe QDs demonstrate that the charging mechanisms in these QDs are influenced by cation intercalation and adsorption. Smaller cations, such as Li^+^, can intercalate into the QD core, enhancing electron injection and radiative lifetime, leading to a remarkable 158% increase in photoluminescence intensity. This intercalation mechanism is critical for LIBs, as it suggests an intrinsic improvement in charge storage efficiency and electron transport dynamics.^53^ Complementing these findings, the exciton radiative lifetime of CdSe QDs and its impact on charge separation at LIB electrodes indicates that smaller QDs exhibit longer exciton lifetimes, which improves charge carrier extraction and collection at the battery electrodes, optimizing overall LIB performance.^54^ The integration of CdSe QDs into Li–O_2_ batteries offers significant advantages in mitigating overpotential and enhancing system stability. One effective approach to enhancing the performance of CdSe QDs is surface passivation techniques, such as encapsulating them with a ZnS shell. This ZnS coating mitigates surface defects, preventing non-radiative recombination that would otherwise degrade the QDs' performance. Incorporating CdSe/ZnS QDs into a CNT network as a photocathode material significantly enhances the efficiency of oxygen reduction and oxidation reactions (ORR/OER) in Li–O_2_ batteries.^27^ The ability of the QDs to generate electron–hole pairs under illumination plays a crucial role in this improvement. Additionally, the QD-CNT hybrid structure facilitates efficient electron transport and enhances the oxidation of Li_2_O_2_, directly addressing the challenge of high overpotential in Li–O_2_ batteries. Since the formation and decomposition of Li_2_O_2_ govern the battery's round-trip efficiency and lifespan, the introduction of surface-passivated CdSe QDs provides a pathway to more reversible electrochemical reactions. This ultimately leads to improved energy density, rechargeability, and stability of Li–O_2_ batteries.

In an investigation conducted on the electrochemical behavior of CdSe QDs, the effects of cation size on charge compensation mechanisms and the resulting optical and electronic modifications were systematically analyzed.^22^ It was found that intercalation of small cations such as Li^+^ led to a 158% enhancement in the band-edge photoluminescence (PL) intensity, alongside the emergence of a strong near-infrared emission peak at approximately 0.76 eV, attributed to the formation of selenium vacancies. In contrast, surface adsorption of bulky cations like TBA^+^ resulted in a more moderate 66% PL increase without the creation of mid-gap states. XPS analysis further confirmed lattice-level changes, revealing shifts in Cd 3d binding energies and the appearance of Cd^0^ and Cd^+^ states only after Li^+^ intercalation. These results underscore the importance of controlled defect engineering and electron injection strategies for optimizing CdSe QDs as active materials in battery applications, where enhanced carrier lifetimes, defect-mediated conduction, and robust electrochemical stability are essential for high-performance energy storage devices.

**Fig. 5:**
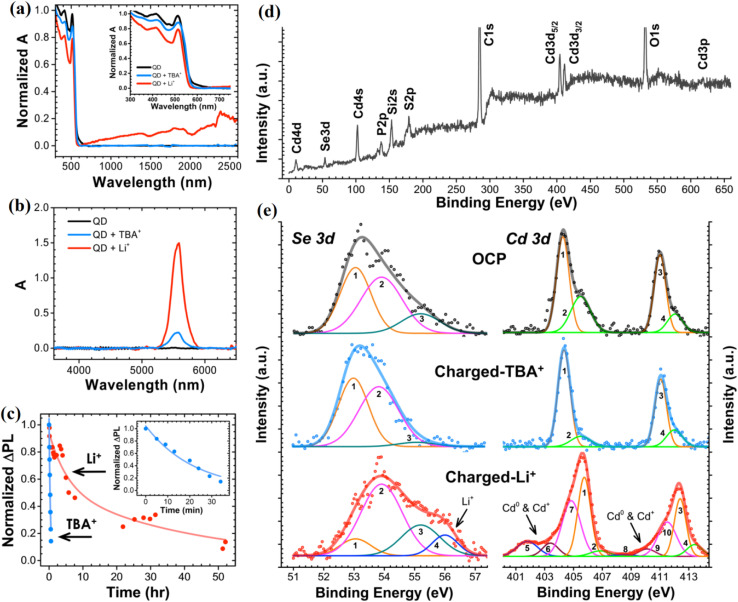
Changes in the optical absorption spectra of CdSe QD in the (a) visible to near-infrared and (b) mid-infrared spectral range as a result of electrochemical charging in Li^+^- and TBA^+^-containing electrolytes. (c) Differences in the kinetics of the decay of photoluminescence intensity of CdSe QD film that was previously charged separately in Li^+^- and TBA^+^-containing electrolytes; X-ray photoemission spectra of CdSe QDs. (d) Survey spectrum of pristine CdSe QDs. (e) Higher-resolution spectra of Se 3d and Cd 3d envelops of pristine (neutral) QDs and QDs charged in TBA^+^- and Li^+^-containing electrolytes. Reproduced with permission from ref. 22. Copyright 2014 American Chemical Society.

Fig. (5a and b) presents the evolution of optical absorption spectra for CdSe QDs during electrochemical charging in the presence of Li^+^ and TBA^+^ ions. Upon charging with Li^+^-containing electrolytes, a broad absorption enhancement is observed across both the visible to near-infrared (600–3000 nm) and mid-infrared (5200–6000 nm) regions, whereas TBA^+^ adsorption mainly increases mid-infrared absorbance without significant visible-NIR changes. This behavior reflects a critical distinction: smaller Li^+^ ions intercalate into the QD lattice and induce lattice-level electronic modifications, while larger TBA^+^ ions are restricted to surface adsorption. In the context of batteries, the ability of CdSe QDs to modify their electronic structure upon ion intercalation suggests their potential as dynamic electrode materials capable of supporting both ionic and electronic conductivity during charge–discharge cycles. Fig. 5(c) provides valuable insight into the kinetics of discharge (self-recovery) of CdSe QDs after electrochemical charging. In TBA^+^-adsorbed systems, photoluminescence (PL) recovery is rapid, with a time constant of approximately 22 minutes, following first-order decay. In contrast, Li^+^-intercalated CdSe QDs exhibit a much slower discharge, characterized by biexponential decay kinetics with time constants of ∼318 and ∼2499 minutes, indicating diffusion-limited Li^+^ deintercalation. For battery applications, this slower discharge behavior implies that Li^+^ intercalation into CdSe QDs could provide a means of stabilizing stored charges over longer periods, thereby enhancing battery shelf-life and suppressing self-discharge losses. Fig. (5d and e) offers critical compositional evidence supporting these mechanisms through X-ray photoelectron spectroscopy (XPS) analysis. Upon Li^+^ intercalation, notable shifts in the Cd 3d peaks toward higher binding energies are observed, along with broadening indicative of Cd^0^ and Cd^+^ species formation. Additionally, a new Se 3d feature emerges around 56 eV, attributed to the formation of Li–Se bonding. These signatures confirm the creation of selenium vacancies (V_Se) and lattice distortions associated with Li^+^ incorporation. In battery systems, such controlled defect generation can enhance ionic transport pathways and modulate electronic properties, leading to improved electrode functionality, greater capacity retention, and better cycling performance.

**Fig. 6:**
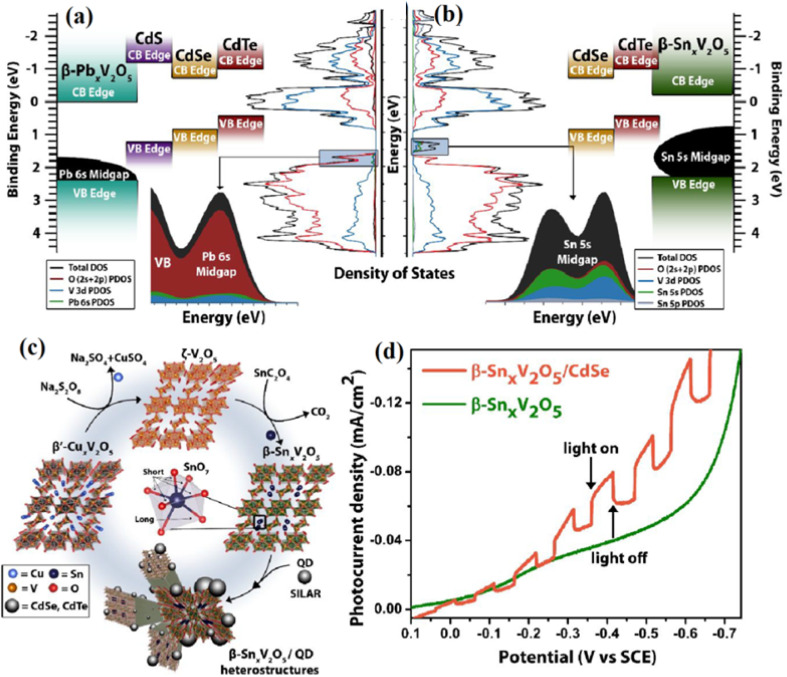
(a and b) Density of states and energy diagrams for CdSe QDs and β-Sn_0_._23_V_2_O_5_, illustrating electronic structure and band alignment. (c) TA spectroscopy showing ultrafast hole transfer from CdSe QDs to β-Sn_0_._23_V_2_O_5_. (d) Photocurrent density showing improved charge storage in β-Sn_0_._23_V_2_O_5_/CdSe QD heterostructures. Reproduced with permission from Reproduced with permission from ref. 55. Copyright 2018 American Chemical Society.

Additionally, the role of CdSe QDs in radioluminescent nuclear batteries has been explored, highlighting their potential for improving energy conversion efficiency.^25^ Incorporating CdSe/ZnS QDs into liquid scintillators optimized spectral matching with photovoltaic devices, leading to enhanced energy transfer and improved battery output performance. In Fig. 6(a), the schematic illustrates the working principle, where beta particles excite the fluorescent layer, generating radioluminescent photons. These photons are absorbed by a photovoltaic module, producing electron–hole pairs that generate electrical current. Fig. 6(b) demonstrates the impact of different concentrations and emission wavelengths (480, 580, and 660 nm) of CdSe/ZnS QDs on the current–voltage characteristics of the battery. The concentrations used in the experiments were 0.5, 1.0, and 4.0 mg mL^−1^ for QDs with emission wavelengths of 480, 580, and 660 nm. The results show that as the concentration of QDs increases, the current also increases, with the highest current observed at 4.0 mg mL^−1^ for the 480 nm wavelength. Similarly, Fig. 6(c) compares the current–voltage characteristics for different fluorescent materials like CsPbBr_3_ perovskite QDs, showing a clear enhancement in performance with optimized material concentrations. Fig. 6(d) focuses on the maximum power output (*P*_max) for various concentrations and emission wavelengths of CdSe/ZnS QDs. The power output for CdSe/ZnS QDs increases significantly, with the highest output achieved at 4.0 mg mL^−1^ concentration for the 660 nm emission wavelength, reaching a *P*_max of approximately 10^−8^ W. Furthermore, integrating gold nanoparticles (Au) with CdSe/ZnS QDs further enhances the radioluminescence efficiency, boosting the battery's output power by up to 15.48 times compared to QDs alone. These findings emphasize the critical role of optimizing both the material properties, such as concentration and emission wavelength, and the structural design to improve the performance of radioluminescent nuclear batteries.

The sensitivity of CdSe/ZnS QDs to gamma radiation—manifested through aggregation and PL quenching—poses significant challenges for their implementation in radioluminescent nuclear batteries.^56^ To address these limitations, protective strategies and structural modifications have been developed to enhance radiation hardness. Encapsulation within robust matrices such as SiO_2_ or Al_2_O_3_ has proven effective, with silica shells (∼5–10 nm thick) preserving 85% of PL intensity after 100 kGy gamma exposure compared to 50% for unencapsulated QDs.^56^ Doping the CdSe core with radiation-resistant elements like indium further improves stability; In-doped CdSe/ZnS QDs retain 80% PL efficiency after 50 kGy irradiation due to enhanced charge carrier recombination.^25^ Polymer-based approaches, including dispersion in poly(methyl methacrylate) (PMMA), mitigate aggregation by maintaining uniform QD distribution and scavenging secondary electrons, yielding 90% PL stability post-10 kGy exposure.^56^ Core–shell optimization, particularly increasing ZnS shell thickness to 2–3 monolayers, reduces surface trap states, improving PL retention by 10–15% under irradiation.^23^ While these advances enable CdSe/ZnS QDs to operate in radiation-intensive environments (*e.g.*, alpha-voltaic batteries^57^), trade-offs include elevated production costs (∼10–20%) from encapsulation and potential alterations in electrochemical properties, necessitating application-specific tuning.^1^ Collectively, these strategies enhance the viability of QDs for nuclear battery systems where stable radioluminescence is critical for photovoltaic energy conversion.

The enhancement of charge transfer between QDs and electrode materials plays a pivotal role in optimizing battery performance, particularly in next-generation energy storage systems. Surface modification of CdSe QDs using conjugated organic ligands has demonstrated a significant improvement in electron transport when applied to TiO_2_-based QD-sensitized solar cells (QDSSCs).^58^ Functionalization with disulfide bonds and benzene ring structures not only stabilized the QDs but also reduced charge recombination, thereby increasing power conversion efficiency. Given that charge transfer limitations are a critical bottleneck in lithium-based batteries, these surface engineering strategies could be adapted to Li-ion and lithium–air (Li–O_2_) batteries to improve interfacial electron kinetics. By mitigating charge recombination and enhancing electron injection, the integration of ligand-modified CdSe QDs into battery electrodes could lead to more efficient charge transport, ultimately enhancing energy density and cycle stability in rechargeable battery systems. Moreover, the study's findings align with the broader goal of optimizing QD-based electrode materials for electrochemical applications.

By leveraging ligand exchange strategies, CdSe QDs can be tuned to exhibit improved charge transfer properties, which is essential for maximizing the redox efficiency in battery systems. The fluorescence quenching and lifetime decay observed in modified CdSe QDs suggest an enhanced electron extraction capability, which could be particularly beneficial in high-performance lithium-ion and Li–O_2_ batteries where rapid charge transfer is crucial. In a related study, surface ligand modifications on CdSe QDs were shown to improve both electron transfer and chemical stability, making the QDs more durable and suitable for long-term energy storage applications.^25^ These improvements are particularly valuable for applications requiring stable performance over extended periods, such as in long-lasting batteries and energy-harvesting devices. CdSe QDs are being increasingly utilized in hybrid energy storage and conversion systems, where their unique optoelectronic properties enhance charge transport and electrochemical stability. For instance, a PbSe/CdSe core–shell QD-based hybrid solar battery with graphene electrodes achieved a conversion efficiency of 3.6%.^59^ This improvement was attributed to enhanced light absorption, efficient charge carrier separation, and reduced internal resistance enabled by the graphene–QD interface. Graphene, as an electrode material, not only reduced sheet resistance but also accelerated electron transport, a crucial factor for both solar-driven energy storage and battery applications. This strategy offers a promising direction for the development of photo-rechargeable batteries, which directly convert and store solar energy in electrochemical systems.

The co-sensitization of CdSe and CdS QDs in QD-sensitized solar batteries (QDSBs) has been investigated, demonstrating that the broadened absorption spectrum and increased photogenerated charge carriers significantly enhanced the overall electrochemical performance.^60^ Optimized anode structures and fabrication techniques could improve charge retention and cycling stability, which are essential for practical energy storage applications. The synergistic effects of CdS and CdSe co-sensitization not only improved power conversion efficiency but also contributed to more stable charge–discharge cycling in photo-assisted energy storage devices. CdSe QDs have been strategically employed in nuclear battery technologies due to their ability to optimize spectral matching and enhance radioluminescence conversion efficiency. Incorporating CdSe/ZnS QDs into liquid scintillators improved radioluminescent nuclear battery performance by precisely tuning the emission spectrum to align with the absorption range of the GaAs photovoltaic device.^61^ This spectral tuning was achieved by controlling the composition and concentration of QDs within the scintillator matrix, ensuring an optimized energy transfer process. CdSe/ZnS QDs facilitated a more efficient radioluminescence process under X-ray excitation, with 15 mg of CdSe/ZnS QDs in Emulsifier-Safe scintillator yielding the highest electric power output. The mechanism underlying this enhancement involved increased radiative recombination efficiency of the QDs, reducing non-radiative losses and improving photon harvesting by the photovoltaic component. Furthermore, spectral matching analysis revealed that higher QD concentrations improved energy transfer efficiency by reducing the mismatch between emitted photons and photovoltaic absorption bands, leading to an overall increase in battery efficiency. In alpha voltaic batteries, CdSe QDs were investigated as radiation-hard intermediate absorbers designed to mitigate direct damage to the semiconductor junction.^57^ The proposed mechanism involved the interaction between high-energy alpha particles and CdSe QDs, where the kinetic energy of the incident alpha radiation was absorbed by the QDs and subsequently re-emitted as visible photons. These photons were then efficiently converted into electrical energy by the photovoltaic cell. By employing CdSe QDs as an intermediary luminescent layer, degradation effects that typically arise when alpha particles directly impact the semiconductor material were reduced, thereby extending battery lifespan and maintaining energy output stability. Additionally, variations in QD composition influenced the emission spectrum, enabling fine-tuned spectral matching with the photovoltaic junction. The study highlighted that integrating QDs within the battery architecture enhanced charge carrier generation efficiency while minimizing non-radiative losses, establishing a pathway for more durable and high-performance alpha voltaic energy sources.

The electroluminescent properties of CdSe QDs have been explored for their potential to enhance energy storage devices by integrating optical–electrical conversion mechanisms within battery architectures. Shen and Guyot-Sionnest (2025) demonstrated mid-infrared electroluminescence in a CdSe QD-based device, where the QDs formed a thin film coupled with ZnO nanocrystals, enabling efficient charge injection and transport.^62^ The ZnO layer played a crucial role in facilitating electron mobility while reducing non-radiative recombination, thereby improving energy transfer within the system. This efficient charge transport mechanism is particularly relevant for advanced battery technologies, where optimizing electron dynamics can lead to improved charge storage and conversion efficiency. The ability of CdSe QDs to sustain stable luminescence under applied voltage suggests potential applications in self-regulating batteries, where electroluminescence could serve as an indicator of charge state or facilitate light-assisted charging processes. Furthermore, incorporating CdSe QDs into organogel matrices has been shown to significantly enhance photoluminescence intensity, with increases up to 528%.^63^ This enhancement is attributed to the suppression of non-radiative decay pathways, as the organogel network provides a stabilized microenvironment that minimizes charge trapping and allows for more efficient exciton recombination. These findings suggest potential applications in energy storage systems, where integrating CdSe QDs into electrolyte materials or solid-state battery components could enhance energy transfer processes, leading to higher performance and longer battery life. CdSe QDs have also demonstrated significant potential in battery-integrated electrochromic energy storage windows (EESWs) by enabling charge-dependent optical modulation. In this context, CdSe QDs were incorporated into a self-powered EESW system, where Prussian blue (PB) acted as a controller of their fluorescence.^64^ This system was powered by a “perpetual” rechargeable battery consisting of Fe/PB and Prussian white (PW)/Pt half-cells, allowing efficient charge storage and self-sustained operation. The electrochemical charging and discharging of the battery directly influenced the fluorescence modulation of CdSe QDs, resulting in a tunable color transition (nonemissive–red–yellow–green) without the need for an external power source. The fabricated EESW exhibited rapid switching times (“off” in 7 s, “on” in 50 s), high transmittance contrast, and a stable fluorescent modulation ratio (60–86%), with minimal performance degradation after 30 cycles. These characteristics highlight the role of CdSe QDs in developing multifunctional battery systems where electrochromic behavior is directly linked to charge storage, enabling real-time visualization of battery states and energy-efficient smart windows. The integration of CdSe QDs in EESWs underscores their applicability in advanced battery technologies by providing both energy storage functionality and interactive optical responses. The rechargeable Fe/PB//PW/Pt battery system facilitates a self-charging mechanism, ensuring continuous electrochromic modulation of CdSe QDs without external bias. This feature not only enhances the efficiency of energy storage systems but also enables the development of self-powered battery interfaces with visual charge indicators. The high sustainability and rapid response of the EESW suggest potential applications in human-readable batteries, real-time energy monitoring, and integrated smart-grid systems. By leveraging the electrochemical interactions between CdSe QDs and rechargeable battery components, this approach opens new avenues for the design of next-generation energy storage devices that combine electrochromic functionality with high-capacity charge retention.

Beyond electrochromic interfaces, a study reveals that CdSe QDs, when hybridized with Cu nanowires (NWs), exhibit enhanced photoluminescence due to the plasmonic and heat transfer effects of Cu NWs.^65^ This enhancement can be leveraged in battery technologies where photoluminescence-assisted charge separation or photon-induced energy conversion is desirable. The improved emission relaxation rate and reduced activation energy of CdSe QDs in such hybrid structures suggest potential applications in optoelectronic battery management systems, light-assisted charging mechanisms, and luminescent charge indicators. By integrating CdSe QDs with advanced battery architectures, their dual role in energy storage and optical signaling could lead to next-generation self-powered and interactive battery technologies, enhancing both efficiency and user interaction in energy storage devices. CdSe QDs serve as efficient photosensitizers in solar cell batteries due to their size-dependent bandgap tunability and high absorption coefficients in the visible spectrum. A study examined the role of QDs in modifying electron transitions within large-bandgap semiconductors, which is critical for optimizing charge separation and transport in solar batteries.^66^ Upon photon absorption, CdSe QDs undergo exciton generation, followed by electron injection into the conduction band of a semiconductor, typically TiO_2_. This process reduces recombination losses and enhances charge carrier lifetimes, directly improving photovoltaic efficiency. Furthermore, localized surface plasmon resonance (LSPR) effects, when CdSe QDs are coupled with metal nanoparticles, can further enhance light harvesting by increasing the effective optical path length within the device.

The latest advancements in CdSe QD-based solar batteries emphasize their integration into flexible architectures to achieve both high efficiency and mechanical durability. A study fabricated a flexible CdS/CdSe QD-sensitized solar battery using a blade-coated ITO/PEN substrate, where controlled interface engineering played a key role in optimizing charge transfer dynamics.^67^ The study demonstrated that the introduction of *tert*-butanol additives significantly improved the binding properties between layers, enhancing the electron injection efficiency and achieving a peak energy conversion efficiency of 3.49%. Moreover, hydrochloric acid additives contributed to the long-term mechanical stability of the device, preserving 72.7% of its efficiency after extensive bending cycles. These results highlight the necessity of precise interfacial engineering and additive-controlled film formation in CdSe QD-based solar batteries to achieve both superior charge transport and structural integrity.

## 5. CdSe QDs in supercapacitors

The integration of QDs into supercapacitor technology has become an area of increasing interest due to their unique properties, including high surface area and tunable electronic characteristics. These properties make QDs ideal candidates for enhancing the electrochemical performance of supercapacitors, especially in terms of charge storage, conductivity, and long-term stability. In particular, the synthesis and surface modification of CdSe-based QDs are crucial for optimizing their performance in such applications. A study developed a non-injection, one-pot synthesis method for CdSeS alloyed QDs, offering a scalable and reproducible process.^68^ By adjusting the Cd/Se/S molar ratios during the synthesis, the optical properties of the QDs, notably the absorption and emission spectra, were controlled. The study showed that a high Cd/Se/S ratio resulted in larger QDs with a smaller bandgap, which is beneficial for improving charge transport and conductivity, essential for supercapacitor electrodes. This approach allowed the creation of QDs emitting in the range of 470–550 nm, a feature that would be challenging to achieve with binary CdS or CdSe QDs alone. The fine-tuning of the bandgap through composition rather than size offers a promising avenue for developing high-performance supercapacitors, as it ensures that the QDs possess optimal electrochemical properties for efficient charge storage and rapid charge/discharge cycles.

Additionally, research has highlighted the importance of surface modification through ligand exchange to enhance the stability and dispersion of CdSe QDs in aqueous electrolytes.^69^ The study revealed that exchanging the original hydrophobic ligands, trioctylphosphine (TOP) and trioctylphosphine oxide (TOPO), with thiolated polyethylene glycol (PEG) polymers significantly improved the dispersion stability of CdSe QDs in polar solvents like water. The surface concentration of both TOP/TOPO and PEG ligands was quantified, showing that the exchange removed 50–85% of the original ligands and replaced them with PEG. Despite PEG accounting for a smaller portion of the surface coverage, it was sufficient to disperse the QDs in polar solvents and improve the interactions between the QDs and the electrolyte. These interactions are critical for supercapacitor performance as they reduce internal resistance and improve charge mobility. Moreover, the modified QDs were no longer dispersible in nonpolar solvents, highlighting the successful transition from a nonpolar to a polar solvent system, which is essential for enhancing the efficiency of supercapacitors in aqueous environments.

Murray *et al.* (2023) provided further insights into the stability of CdSe/ZnS QDs under ionizing radiation, which is important for the long-term reliability of supercapacitors.^56^ They exposed CdSe/ZnS QDs to gamma radiation and monitored the changes in their photoluminescent properties. Their study found that exposure to increasing doses of gamma radiation led to the degradation of certain photoluminescent components. The study revealed that radiation-induced degradation was more pronounced when samples had larger air headspaces, which accelerated the formation of larger QD aggregates, negatively impacting charge transport and electrochemical performance. These findings are particularly relevant for supercapacitors that may be subjected to harsh environmental conditions, such as radiation or extreme temperatures, suggesting that while CdSe/ZnS QDs are resilient, controlling aggregation and environmental exposure is crucial for maintaining performance in the long term. A promising approach to improving the properties of CdSe-based materials is through bandgap engineering, such as the creation of core–shell or alloyed QDs. Recent studies have explored the synthesis of CdTe/CdSe core–shell QDs and CdTeSe alloy QDs, revealing significant tunability in their optical and electrochemical characteristics.^70^ Bandgap tuning, achieved by controlling shell thickness and doping elements, not only alters emission spectra but also impacts charge transport and energy storage. Core–shell CdTe/CdSe QDs showed a red shift in emission maximum (EM) up to 125 nm and a corresponding increase in Stokes shift, indicating enhanced electron–hole separation. These optical features are crucial for photoelectrochemical capacitors, where efficient charge separation is key for high energy density and fast charge–discharge cycles. The increase in emission lifetime (53 ns compared to CdTe) in CdTe/CdSe QDs further supports improved charge storage, essential for supercapacitors, where cycling stability and efficient electron transfer are vital. In contrast, the defect state in CdTeSe alloy QDs decreased the lifetime by 2 ns, suggesting that defect engineering can modulate charge dynamics, potentially benefiting supercapacitors in high power density applications. The core–shell structure and alloyed QDs provide unique opportunities to optimize electrochemical performance. For instance, the double band-edge exciton absorption in CdTe/CdSe QDs (peaks at 354–373 nm and 401–436 nm) signifies wave function separation between core and shell. This characteristic can enhance capacitance retention and cycling stability in supercapacitors by improving charge storage and reducing charge recombination, making these materials ideal for advanced energy storage applications.

The performance of supercapacitors is largely determined by the charge storage mechanism and electrode design, where CdSe QDs have emerged as promising materials due to their tunable properties and high surface area. CdSe QDs, when deposited on transparent ITO substrates, exhibit capacitive behavior in acidic electrolytes, with red CdSe QDs displaying more than ten times higher specific capacitance than their orange counterparts. This significant difference highlights the crucial role of QD size and bandgap in electrochemical performance, influencing charge transfer efficiency and interaction with electrode materials. Additionally, red CdSe QDs showed superior cycling stability and low internal resistance, making them suitable for high-performance energy storage applications.

**Fig. 7:**
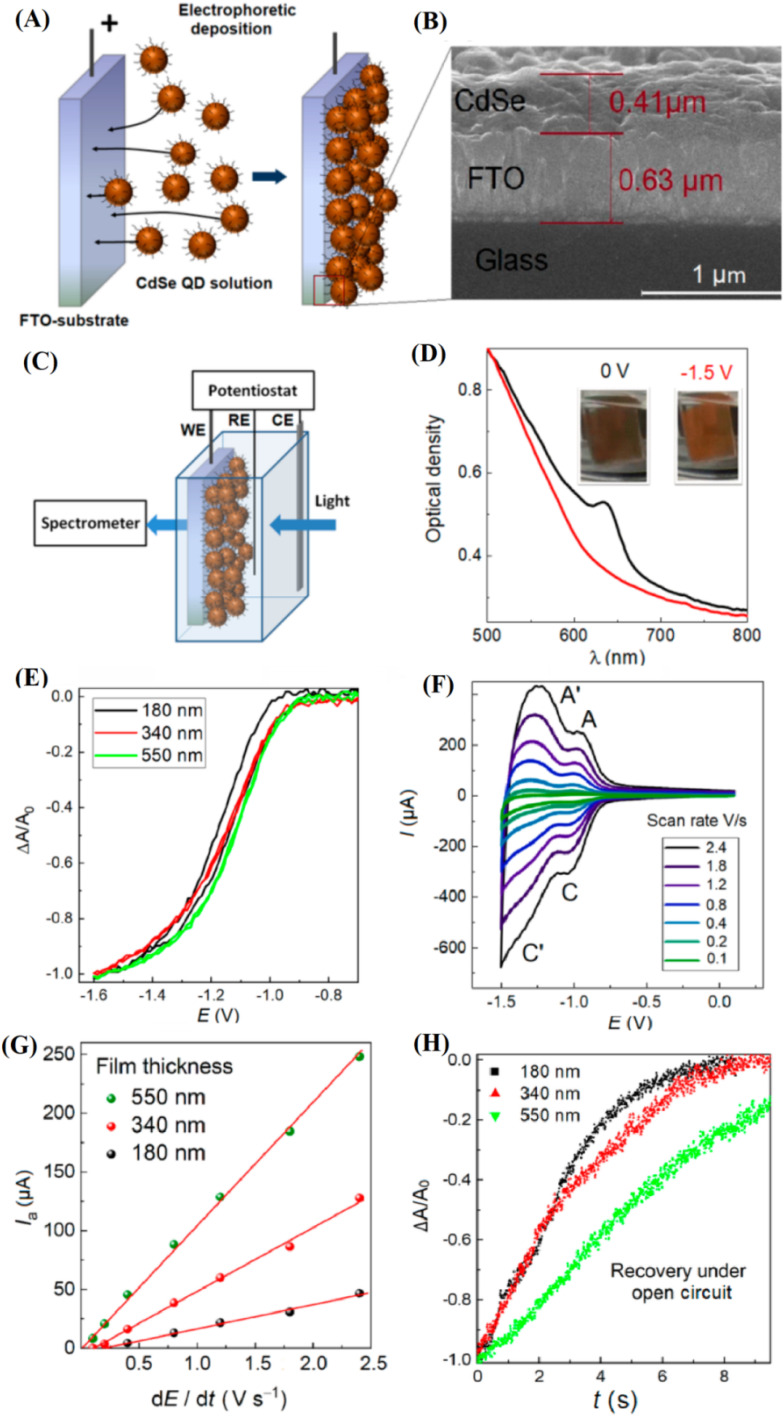
(A) Schematic of electrophoretic deposition of CdSe QDs onto an FTO substrate. (B) Cross-sectional SEM image showing a 0.41 μm thick CdSe QD layer on a 0.63 μm thick FTO substrate. (C) Electrochemical measurement setup, including a potentiostat for voltage application and a spectrometer for absorption measurements. (D) Optical density variations at 0 V and −1.5 V, demonstrating electrochromic behavior of CdSe QDs. (E) Cyclic voltammetry data showing redox activity at different film thicknesses (180, 340, and 550 nm) of CdSe QDs. (F) Cyclic voltammogram demonstrating redox peaks confirming efficient charge storage in CdSe QDs. (G) Film thickness *vs.* current density and scan rate, showing improved charge transport in thinner CdSe QD films. (H) Charge recovery under open-circuit conditions, demonstrating faster recovery in thinner CdSe QD films (∼10 s). Reproduced with permission from ref. 72. Copyright 2021 American Chemical Society.

Expanding on the electrodeposition process, researchers optimized the deposition time of CdSe QDs and evaluated their electrochemical performance.^71^ Their study found that CdSe films deposited for 40 minutes achieved an outstanding specific capacitance of 1542 mF cm^−2^ at a current density of 1 mA cm^−2^, with capacitance retention of 78.9% after 1000 cycles. This performance highlights the potential of CdSe as a viable material for durable supercapacitor electrodes, offering both high capacitance and long-term stability. Moreover, the study also demonstrated the practicality of CdSe-based electrodes by assembling symmetrical aqueous and solid-state supercapacitors, with both configurations capable of lighting up an LED. These results reinforce the promise of CdSe in supercapacitor applications, showcasing both its high energy storage capacity and its applicability in real-world energy storage devices. Additionally, another study explored electrophoretically deposited (EPD) CdSe QD films, focusing on their electrochromic and electrochemical properties.^72^ The findings demonstrated that the EPD CdSe QD films exhibited fast charge–discharge kinetics, with complete absorption bleaching and recovery occurring in less than 100 milliseconds. This rapid switching ability, along with their stable electrochromic response, opens up new possibilities for CdSe QD films in next-generation supercapacitors, especially for applications where fast charging and discharging are required. The films showed a strong and reversible electrochromic response, which is crucial for applications such as electrochromic displays and smart windows, where both optical and electrochemical performance are needed. Fig. 7(A) depicts the electrophoretic deposition of CdSe QDs onto an FTO substrate, forming a uniform layer with controlled thickness. This uniform deposition ensures efficient charge storage, which is essential for achieving a high specific capacitance, typically in the range of 100–300 F g^−1^, depending on material composition and structural optimization. Fig. 7(B) presents a cross-sectional SEM image, confirming the formation of a 0.41 μm thick CdSe QD layer on a 0.63 μm thick FTO substrate. A well-structured CdSe layer minimizes charge recombination and enhances conductivity, leading to improved electrochemical performance. The interaction between CdSe QDs and the FTO substrate ensures low internal resistance, with impedance values typically below 10 Ω, facilitating efficient charge storage and retrieval. Such improvements contribute to the overall energy density of the supercapacitor, which can reach up to 10 W h kg^−1^ in optimized conditions.

Fig. 7(C) outlines the electrochemical measurement setup, where a potentiostat applies voltages while a spectrometer measures absorption changes. The setup helps evaluate charge injection and extraction processes, which directly impact charge/discharge efficiency. Efficient charge transport in CdSe QDs enables fast response times, with charge transfer rates often exceeding 10^6^ s^−1^, crucial for high-power-density supercapacitors. Lower charge transfer resistance (*R*_ct_ < 50 Ω) further improves charge mobility, reducing energy losses during operation. Fig. 7(D) shows optical density variations at 0 V and −1.5 V, demonstrating electrochromic behavior linked to charge accumulation. The redox peaks observed around −1.4 V confirm efficient charge storage, with current densities reaching 200–300 μA cm^−2^, reinforcing the suitability of CdSe QDs for fast and reversible energy storage applications. Fig. 7(G) shows that thinner films (180 nm) have higher current densities and faster response times than thicker films (550 nm), indicating better charge transport efficiency. Fig. 7(H) highlights charge recovery under open-circuit conditions, where thinner films recover charge within ∼10 s, reducing recombination losses and enhancing charge retention. These findings confirm that CdSe QDs provide an excellent platform for high-performance supercapacitors, balancing energy density (∼10 W h kg^−1^) and power density (∼10 kW kg^−1^) for advanced energy storage applications.

Taken together, these studies illustrate the significant role of CdSe QDs in enhancing supercapacitor performance. Research has demonstrated that the size and color of CdSe QDs can directly influence capacitance, while optimization of the deposition process has led to high capacitance and stability over long cycling periods. Furthermore, studies have highlighted the fast charge–discharge kinetics and electrochromic potential of CdSe QD films^71–73^. These findings underscore the importance of both the synthesis and the surface modification of CdSe QDs in developing high-performance, durable, and versatile supercapacitors. The ongoing refinement of CdSe QD properties, including size, surface modification, and deposition techniques, promises to further enhance the electrochemical performance and practical applications of supercapacitors based on QD technology.

The development of hybrid nanostructures incorporating CdSe QDs has emerged as a promising strategy for improving charge storage efficiency in supercapacitors. Hybrid materials combine the unique properties of different nanomaterials, enhancing electrochemical performance by optimizing charge transport, ion diffusion, and cycling stability. Shah *et al.* (2023) introduced a NiSe_2_/CdSe hybrid electrode that achieved an impressive capacitance of 358C g^−1^ and demonstrated enhanced long-term stability.^33^ This composite structure facilitated fast ion diffusion by reducing charge transfer resistance, making it a suitable candidate for high-performance supercapacitors. The optimized electrochemical properties of this hybrid material were reflected in its outstanding energy storage performance, with a hybrid supercapacitor demonstrating a high energy density of 40.5 W h kg^−1^, maximum power of 7161.2 W kg^−1^, and excellent cycling stability over 15 000 cycles. This approach highlights the potential of combining CdSe with other materials to significantly improve the efficiency and durability of energy storage devices. Similarly, CdSe@PbS core–shell nanostructures have been explored for supercapacitor applications.^74^ This design utilized a simple, low-cost chemical method to fabricate CdSe nanowires as templates, followed by ion exchange to form CdSe NWs, which were then encapsulated with PbS nanoparticles *via* the successive ionic layer adsorption and reaction (SILAR) method. The resulting core–shell structure not only provided maximum active sites but also facilitated smooth and rapid electron transfer, leading to a specific capacity of 82 mA h g^−1^ and a notable energy density of 16.14 W h kg^−1^ at a power density of 288 W kg^−1^. The hybrid structure demonstrated enhanced electrochemical performance, including improved long-term stability, making it an attractive candidate for high-performance supercapacitors. The design of this hybrid structure, with its efficient electron transport pathways and large active surface area, offers a promising strategy for enhancing the energy storage capabilities of QD-based materials.

In a different approach, CdSe-based heterostructures interfacing with vanadium oxide have been designed, demonstrating the importance of ultrafast hole extraction in enhancing charge separation and storage efficiency.^55^ These heterostructures were specifically tailored to promote light-induced charge separation, with the interfacial electronic structure facilitating ultrafast subpicosecond hole transfer from CdSe QDs to the β-Sn_0_._23_V_2_O_5_ component. This efficient hole extraction and rapid charge separation are critical for enhancing photocatalytic processes and improving the overall performance of energy storage devices. Although the focus of Andrews *et al.*^55^ was on photocatalytic applications, the principles of fast charge transfer and efficient hole extraction are directly relevant to supercapacitors, where similar mechanisms can be employed to optimize charge storage and reduce internal resistance in hybrid nanostructures.

Surface functionalization has emerged as a key strategy for enhancing the electrochemical properties of CdSe QDs, optimizing their performance in energy storage applications. Fe-doped CdSe QDs have been synthesized, where the incorporation of iron reduced the bandgap energy, enhancing conductivity and significantly increasing the areal capacitance from 47 mF cm^−2^ to 73 mF cm^−2^.^75^ This modification allowed for better charge storage and transfer, making the material more efficient for use in electrochemical capacitors. The incorporation of metal dopants like Fe is a well-known technique for tuning the electronic properties of semiconducting QDs, leading to improved charge storage capabilities and increased capacitance, which are critical for developing high-performance supercapacitors. Similarly, a green synthesis method using kappa-carrageenan (κ-CGN) has been employed to prepare CdSe QDs for use in electrochemical capacitors.^76^ By incorporating CdSe QDs into an activated carbon (AC) matrix, the resulting AC@CdSe composite exhibited significantly enhanced electrochemical properties. The specific capacitance of the AC@CdSe electrode was found to be 480 F g^−1^, compared to 103 F g^−1^ for the AC-only electrode. This composite also displayed an energy density of 52 W h kg^−1^ and a power density of 2880 W kg^−1^, with excellent cycling stability, maintaining high capacitance over 10 000 cycles. The green synthesis approach, coupled with the use of non-toxic materials like κ-CGN, not only enhanced the electrochemical performance of the supercapacitor but also made the process environmentally friendly, offering a sustainable solution for energy storage applications.

A study also examined a composite material combining CdSe QDs with MoS_2_ nanosheets, enhancing electrochemiluminescence (ECL) and ion diffusion properties. In supercapacitors, these improvements boost charge storage and energy release efficiency. MoS_2_ nanosheets provide a conductive matrix, improving ionic diffusion and charge transport within electrodes, while CdSe QDs increase charge storage by offering more active sites.^77^ This synergy ensures efficient charge/discharge cycles, essential for high-performance supercapacitors. MoS_2_ aids electron conduction, while CdSe QDs enhance electrochemical stability and energy storage through quantum confinement. The composite's ECL reveals electron transfer mechanisms, with MoS_2_ stabilizing CdSe QDs, preventing oxidation, and promoting the formation of charged species (CdSe^−^˙) during electrochemical reactions. This stability is crucial for supercapacitors, where minimal degradation during cycling is essential. As a result, the MoS_2_-CS/CdSe QDs composite improves charge transfer properties, long-term stability, and efficiency, potentially leading to supercapacitors with higher capacitance, faster charge/discharge rates, and greater cycling stability than conventional materials. The TEM images in Panels 8A and 8B show the morphology of MoS_2_ (A) and CdSe QDs (B), which are crucial for enhancing the electrochemical properties of supercapacitors. The size and distribution of CdSe QDs significantly impact the charge storage capacity and energy release efficiency in supercapacitor electrodes. The MoS2 nanosheets provide a conductive matrix to support the QDs, improving charge transport, while chitosan stabilizes the composite, ensuring better long-term performance. In Panel 8C, the cyclic voltammogram (CV) illustrates the electrochemical response of the MoS_2_-CS/CdSe QDs composite, showing how the QDs influence charge transfer and capacitance. Panels 8D and 8E highlight how the applied potential and scan rate affect the electrochemiluminescence (ECL) intensity, which is directly related to the charge/discharge cycles in supercapacitors. Panel F further confirms how the composite's electrochemical behavior optimizes the overall performance, with the CdSe QDs playing a key role in enhancing capacitance and power output in energy storage applications.

In the pursuit of improving the stability and performance of supercapacitors, a critical challenge lies in ensuring long-term cycling stability and enhancing energy storage capabilities. A study investigated the effect of varying CdSe deposition times on the morphology and electrochemical performance of CdSe electrodes.^78^ Through experiments, it was found that by extending the deposition time, the morphology of the CdSe thin films became less uniform, leading to the growth of CdSe microspheres with a higher size and density. This change in morphology resulted in a significant improvement in the a real capacitance, which increased from 0.30 mF cm^−2^ to 1.285 mF cm^−2^. These films exhibited not only improved electrochemical performance but also high stability over extended charge–discharge cycles, demonstrating the positive impact of deposition time on the overall electrochemical performance. The enhancement in capacitance and stability is essential for supercapacitors to achieve reliable, long-lasting energy storage capabilities, making these CdSe-based electrodes a promising candidate for energy storage applications. In a similar vein, Wassel *et al.* (2024) focused on doping-dependent structural, optical, and electrical changes in CdSe thin films, specifically by incorporating indium into the CdSe structure.^79^ The indium-doped CdSe films exhibited enhanced electrical conductivity, as well as improved optical properties, leading to significantly better photodetection performance. The highest electrical conductivity was achieved with 37.5 wt% indium doping, reflecting a strong correlation between the doped films' structural and optical characteristics and their photodetection performance. These doped films were integrated with p-type silicon to form heterojunctions, which showed promising photodetection capabilities with a photocurrent density of 34.4 mA cm^−2^, a high external quantum efficiency (EQE) of 800%, and rapid response times of 3.06 ms for rise time and 6.10 ms for fall time. The remarkable photodetection performance of these indium-doped CdSe films indicates their potential for applications in self-monitoring supercapacitors, where enhanced photodetection could provide real-time feedback on the performance and health of energy storage devices.

Furthermore, Rawat *et al.* (2023) synthesized CdSe–TiO_2_ nanocomposites, aiming to improve both photocatalytic activity and energy storage efficiency.^35^ By integrating CdSe QDs with titanium dioxide (TiO_2_) nanoparticles, they created a hybrid material that exhibited excellent photocatalytic degradation of methylene blue (MB) dye. The combination of CdSe's ability to absorb visible light and TiO_2_'s photocatalytic properties led to enhanced photocatalytic performance. The photocatalytic performance of the CdSe–TiO_2_ nanocomposites showed significant dye degradation, with potential applications in environmental remediation. Additionally, the study suggested that these nanocomposites could be used in dual-purpose applications, such as photo-supercapacitors, where the CdSe–TiO_2_ hybrid material could serve as both an energy storage device and a photocatalyst. This dual-functionality makes the material particularly attractive for sustainable energy solutions, as it allows for simultaneous energy storage and environmental cleanup. These studies underline the versatility of CdSe-based materials for supercapacitor technology. By optimizing the deposition time, incorporating doping strategies, and developing hybrid materials like CdSe–TiO_2_ nanocomposites, researchers have made significant strides in enhancing the electrochemical and photocatalytic properties of CdSe. Such advancements not only improve energy storage capabilities but also open new avenues for multifunctional devices that can serve both energy storage and environmental remediation purposes.

The hybridization of CdSe QDs with materials such as graphene, carbon nanotubes, perovskites, and core–shell QDs has led to significant improvements in the electrochemical performance of supercapacitors. These hybrid materials offer enhanced charge storage, improved cycling stability, faster charge–discharge times, and higher power densities. Furthermore, the application of CdSe QDs in photoelectrochemical and electrochemiluminescent devices demonstrates their multifunctionality, opening new avenues for the development of self-powered energy storage systems and highly sensitive sensors.^79^ One of the most significant contributions to the electrochemical performance of CdSe-based supercapacitors comes from the hybridization with sulfur and nitrogen co-doped graphene QDs (S,N-GQDs). A study synthesized CdSe/S,N-GQDs composites and evaluated their electrochemical properties in a three-electrode configuration.^80^ The results showed a remarkable increase in photocurrent density, reaching 4.286 × 10^−5^ a cm^−2^ under UV light (365 nm) irradiation, which was 10.5 times higher than that of pure CdSe QDs. This enhancement is attributed to the role of S, N doping, which promotes the separation of photogenerated charge carriers. The increase in specific surface area due to the presence of graphene also enhances electron transfer, thus improving the electrochemical capacitance. These findings suggest that the hybrid system's improved charge carrier dynamics make it a highly efficient candidate for supercapacitor applications where fast charge–discharge cycles are critical. In a different approach, CdSe QDs were integrated with organometallic halide perovskites to fabricate thin-film electrochemical capacitors.^81^ This integration demonstrated long-term cycling stability, with stable capacitance outputs exceeding 4000 charge–discharge cycles. The impedance spectroscopy results revealed that perovskites not only serve as active electrodes but also as solid electrolytes, which reduces the charge transfer resistance and enhances the overall energy density of the supercapacitor. The high surface area of the hybrid structure facilitates better electrolyte accessibility to the active electrode materials, further improving the device's electrochemical performance. Additionally, the low relaxation time measured through impedance analysis suggests that these hybrid capacitors could potentially exhibit fast charging capabilities in real-world applications.

The combination of CdSe QDs with CNTs provides significant electrochemical improvements, particularly for supercapacitors. The hybrid material, CdSe@CNT, demonstrated enhanced UV-vis absorption and photoluminescence properties, indicating its potential for optoelectronic applications.^82^ For supercapacitor performance, the addition of CNTs to CdSe QDs contributes to improved electrical conductivity, creating a more efficient path for electron transfer. This is crucial for supercapacitors, as the CNTs facilitate faster electron movement, which enhances the charge/discharge cycles and overall power density. The increased conductivity, combined with the high charge storage capacity of the CdSe QDs, allows the hybrid material to store more energy while ensuring rapid energy release during operation. In supercapacitors, the synergy between CdSe QDs and CNTs plays a vital role in optimizing both energy storage and electron transport. The CdSe QDs provide a high surface area for charge accumulation, while the CNTs improve the electron conductivity, ensuring that electrons are transferred quickly during charge/discharge cycles. This hybrid structure allows for high power density, longer cycle life, and better overall electrochemical performance, making it a promising material for next-generation supercapacitors.

Moreover, the incorporation of CdS@CdSe core–shell QDs improves the photoelectrochemical properties of CdSe, which is particularly important for self-powered supercapacitors. The synthesis of CdS@CdSe core–shell QDs and their photoelectrochemical behavior have been investigated.^83^ The current densities observed for the core–shell structure were significantly higher than those of individual CdSe or CdS QDs, primarily due to the built-in electric field at the CdS/CdSe interface. This electric field promotes the efficient separation of photogenerated charge carriers, leading to enhanced charge collection efficiency and faster electron transfer. As a result, these core–shell QDs exhibit superior photoresponsivity and stability under ambient conditions, making them ideal candidates for renewable energy applications in addition to energy storage. The use of CdSe QDs in electrochemiluminescence (ECL) immunosensors for environmental monitoring has been demonstrated.^84^ Graphene nanosheets were used as a scaffold to capture CdSe QDs, resulting in a significant enhancement of the ECL signal—about 7 times higher than that of the pure CdSe QDs-based probe. This improvement can be attributed to the increased number of active sites provided by the MoS_2_–Au hybrid nanocomposite matrix, which facilitates better electron transfer between the electrode and the CdSe QDs. The ECL immunosensor developed using this hybrid material exhibited a detection limit of 0.0032 μg L^−1^ for microcystin–leucine–arginine (MCLR), showing excellent selectivity and reproducibility. These findings highlight the versatility of CdSe QDs not only in energy storage but also in highly sensitive electrochemical detection systems.

## 6. Compare mechanisms of CdSe QDs in batteries and supercapacitors

CdSe QDs exploit their nanoscale properties—high surface area, tunable bandgap, and redox activity—to enhance electrochemical performance in batteries and supercapacitors. This section provides a detailed comparison of these mechanisms, focusing on their electrochemical contributions.

### 6.1. Surface area and charge dynamics

CdSe QDs, sized 2–10 nm, possess a high surface-to-volume ratio exceeding 100 m^2^ g^−1^, significantly increasing active sites for electrochemical reactions.^85–87^ In LIBs, this facilitates efficient Li^+^ intercalation, achieving specific capacities up to 700 mA h g^−1^ at a 10C rate (full charge in 6 minutes).^88^ The nanoscale dimensions reduce ion diffusion lengths, enhancing reaction kinetics critical for fast-charging applications like electric vehicles (EVs).^89–93^ Time-resolved photoluminescence (TRPL) studies reveal ultrafast electron transfer with decay times of 0.5–7 ns,^24^ accelerating charge extraction and improving capacity retention. This dynamic is driven by quantum confinement, which confines charge carriers, boosting their mobility compared to bulk materials. In supercapacitors, the expansive surface area enhances electric double-layer capacitance (EDLC) through rapid ion adsorption, delivering specific capacitances of 400 F g^−1^ and power densities of 10 kW kg^−1^.^56^ This high power output suits applications requiring instant energy bursts, such as regenerative braking. The shortened diffusion paths ensure swift ion transport, a key advantage over traditional carbon-based electrodes with larger particle sizes.

The dual electrochemical mechanisms of CdSe QDs—electric double-layer capacitance (EDLC) and pseudocapacitance—enable high specific capacitances (∼400–600 F g^−1^) and power densities (10 kW kg^−1^) in supercapacitors, but their deployment in large-scale applications like electric vehicles (EVs) and renewable energy grid storage faces significant practical challenges.^59,71^ Scalability is hindered by the complex synthesis of CdSe QDs, such as hot-injection methods requiring high temperatures (300–350 °C) and inert atmospheres, which increase production costs compared to activated carbon.^1,24^ Microwave-assisted synthesis offers a faster, more scalable alternative, producing uniform QDs in minutes, but specialized reactors limit widespread adoption.^48^ Reproducibility is challenged by size distribution variability, particularly in hot-injection synthesis (*σ* ≈ 15–20%), leading to capacitance fluctuations of 10–15% across batches, which complicates quality control for large-scale manufacturing.^22^ Room-temperature synthesis, with tighter size control (*σ* ≈ 5–7%), improves reproducibility but yields lower pseudocapacitive performance.^30^ Real-world performance challenges include thermal instability under high-rate cycling in EVs, where localized heating degrades QD structure, reducing capacitance retention to 80% after 5000 cycles compared to activated carbon's 95%.^70^ In grid storage, electrolyte incompatibility with CdSe QDs can form insulating layers, increasing impedance and lowering efficiency (∼90% *vs.* 95% for carbon-based systems).^63^ The high surface area (>100 m^2^ g^−1^) enhances EDLC, but pseudocapacitance relies on redox stability, which is sensitive to electrolyte pH and cycling conditions, necessitating tailored electrolytes or protective ZnS coatings.^23^ Additionally, cadmium's toxicity imposes regulatory constraints, requiring costly encapsulation and recycling processes, unlike non-toxic metal oxides.^1^ Compared to emerging technologies like graphene-based supercapacitors (500–700 F g^−1^, 95% retention), CdSe QDs offer competitive power density but lag in scalability and cost. Strategies like cadmium-free ZnSe QDs and automated synthesis could enhance feasibility, but significant investment in production infrastructure is needed to address these challenges for practical large-scale deployment.^24,48^ Studies indicate that the high surface area also promotes uniform charge distribution, reducing internal resistance and enhancing overall efficiency.^80^ The interplay between surface area and charge dynamics bridges the gap between energy-focused batteries and power-oriented supercapacitors, making CdSe QDs a versatile material. Their ability to optimize ion and electron movement underpins their potential in next-generation energy storage systems, from portable electronics to grid-scale solutions, where both capacity and speed are paramount.

### 6.2. Pseudocapacitance

CdSe QDs exhibit pseudocapacitance through reversible faradaic redox reactions involving Cd^2+^/Se^2−^ transitions, significantly enhancing charge storage beyond traditional mechanisms. In batteries, this behavior drives high energy density, with sodium-ion systems achieving 500 mA h g^−1^*via* surface redox processes.^54^ Additionally, conversion reactions (*e.g.*, CdSe + 2Li^+^ → Cd + Li_2_Se) in LIBs push capacities to 900 mA h g^−1^,^61^ surpassing conventional intercalation materials like graphite. These reactions involve bulk phase transformations, making them ideal for energy-intensive applications such as grid storage or EV batteries requiring sustained power output. The nanoscale structure of CdSe QDs amplifies redox site availability, enabling efficient electron injection during cycling.^54^ In supercapacitors, pseudocapacitance complements EDLC, with graphene–CdSe composites reaching 550 F g^−1^.^70^ Here, charge storage is surface-confined, prioritizing rapid electron transfer over deep intercalation, which supports high-power needs like pulsed electronics. To distinguish the dominant charge storage mechanism in CdSe QD-based systems, the contributions of pseudocapacitance and EDLC vary by device configuration. In supercapacitors, CV analysis using the power-law relationship *i* = *av*^*b*^ (where *i* is current, *v* is scan rate, and *b* is the slope) reveals *b*-values of ∼0.8–0.9 for graphene–CdSe QD composites, indicating a dominant pseudocapacitive contribution due to surface redox reactions (*e.g.*, Cd^2+^/Se^2−^ transitions). In contrast, EDLC dominates in systems with larger QDs (>6 nm) and high-surface-area carbon matrices, where *b*-values approach 1.0, reflecting surface-limited ion adsorption.^70^ For LIBs, equivalent circuit modeling from EIS shows low charge transfer resistance (*R*_ct_ ∼50–100 Ω) in CdSe QD electrodes, driven by faradaic conversion reactions (*e.g.*, CdSe + 2Li^+^ → Cd + Li_2_Se), confirming pseudocapacitance as the primary mechanism over EDLC.^61^ These analyses clarify that pseudocapacitance prevails in smaller QDs (<4 nm) and hybrid composites due to abundant redox sites, while EDLC contributes significantly in larger QDs with high surface area, enabling tailored designs for specific energy storage applications.

The redox activity stems from the QDs' high surface area and quantum-confined electronic states, allowing dynamic tuning of charge storage mechanisms. Unlike static bulk materials, CdSe QDs offer flexibility in electrochemical responses, tailoring performance to specific needs.^61^ Research highlights that pseudocapacitive contributions increase with smaller QD sizes due to enhanced surface-to-volume ratios, improving faradaic efficiency.^54^ This dual role—bulk conversion in batteries and surface redox in supercapacitors—positions CdSe QDs as a transformative material. Their ability to augment capacitance and capacity through redox processes addresses key limitations in energy storage, offering a balance of high energy and fast power delivery. This adaptability underscores their potential in hybrid systems, where both energy density and rapid response are critical for advanced applications.

### 6.3. Tunable bandgap

Quantum confinement in CdSe QDs enables bandgap tuning from 1.7 to 2.5 eV by adjusting their size (2–10 nm), optimizing electronic properties for energy storage. In batteries, a narrower bandgap (*e.g.*, 1.7 eV) enhances electrical conductivity, reducing charge transfer resistance and improving rate capability in LIBs.^60^ This facilitates rapid charge–discharge cycles, essential for high-power applications like fast-charging EVs or grid buffers. Smaller QDs with wider bandgaps (*e.g.*, 2.5 eV) provide electrochemical stability, while larger ones prioritize electron mobility, offering tailored responses to redox processes.^60^ In supercapacitors, this tunability accelerates charge–discharge kinetics, achieving superior rate performance at 1.7 eV.^35^ This is critical for rapid energy release in applications such as portable devices or grid stabilization, where power density is paramount. Unlike bulk semiconductors with fixed bandgaps, CdSe QDs' size-dependent electronic structure provides unmatched flexibility, adapting to specific electrochemical demands.^35^ The tunable bandgap influences redox potentials, amplifying pseudocapacitive contributions by increasing electron availability for faradaic reactions.^60^ Studies show that smaller QDs enhance stability under high-rate conditions, while larger ones boost conductivity, optimizing charge transfer efficiency.^35^ This adaptability allows precise matching of QD properties to device requirements, from high-energy storage to high-rate power delivery. The interplay between size and bandgap also affects ion interactions, such as Li^+^ intercalation in batteries, enhancing charge storage through electron injection.^47^ This dynamic tunability positions CdSe QDs as a frontier material, surpassing traditional electrodes in versatility and performance across diverse electrochemical environments, making them ideal for next-generation energy storage systems requiring customized electronic behavior.

### 6.4. Synergistic composites and mechanisms of hybrid stability

Hybridizing CdSe QDs with conductive matrices like graphene or CNTs creates synergistic effects, elevating electrochemical performance in energy storage. In LIBs, graphene–CdSe composites achieve specific capacities of 800 mA h g^−1^, enhancing conductivity and structural stability during cycling. The conductive network shortens Li^+^ diffusion pathways, boosting rate performance and mitigating standalone QDs' low intrinsic electron mobility.^33^ This makes them suitable for high-energy applications like grid storage or EV batteries, where consistent charge transfer is critical. In supercapacitors, similar composites deliver 600 F g^−1^,^35^ improving charge transfer kinetics and capacitance retention over thousands of cycles. CdSe QDs offer distinct performance advantages over conventional electrode materials in LIBs and supercapacitors, but their metrics must be critically compared to assess commercial viability. In LIBs, graphene–CdSe QD composites achieve specific capacities of ∼800 mA h g^−1^ at 0.5C, surpassing graphite anodes (372 mA h g^−1^) and competing with LiCoO_2_ cathodes (∼550 mA h g^−1^).^33^ This high capacity stems from CdSe's tunable bandgap (1.7–2.5 eV) and nanoscale size, which enhance Li^+^ intercalation and electron injection *via* conversion reactions (*e.g.*, CdSe + 2Li^+^ → Cd + Li_2_Se).^61^ However, graphite retains 95% capacity after 1000 cycles, while CdSe QD composites achieve 80–85% retention, reflecting challenges with volume expansion during cycling.^34^ LiCoO_2_ offers similar stability (90% after 500 cycles) but lower rate capability due to slower ion diffusion compared to CdSe's high-rate performance (700 mA h g^−1^ at 10C).^22^ In terms of energy efficiency, CdSe QD-based LIBs achieve round-trip efficiencies of ∼85–90%, slightly below graphite's 90–95% due to higher charge transfer resistance, but their photo-assisted capabilities boost efficiency under illumination by ∼20%.^25^ In supercapacitors, CdSe QD-CNT composites deliver specific capacitances of ∼600 F g^−1^, comparable to activated carbon (∼500–600 F g^−1^) but below metal oxides like MnO_2_ (∼1000 F g^−1^).^35^ CdSe QDs retain 90% capacitance after 10 000 cycles, outperforming MnO_2_ (80% retention) due to their robust nanoscale structure, though activated carbon maintains 95% retention.^70^ Energy efficiency in CdSe QD-based supercapacitors (∼90%) is competitive with activated carbon (∼95%) but benefits from faster charge transfer kinetics.^35^ Despite these advantages, CdSe QDs face challenges in cost and scalability compared to mature materials like graphite, which benefit from established manufacturing. These comparisons highlight CdSe QDs' potential as high-performance alternatives, particularly for high-rate and photo-assisted applications, but underscore the need for improved cycling stability and cost-effective synthesis to compete with commercial systems.

The matrix prevents QD aggregation, ensuring uniform charge distribution and mechanical resilience, which is vital for high-power uses like regenerative braking.^35^ The synergy leverages CdSe QDs' high surface area with the robust conductivity of carbon-based materials, overcoming limitations of pure QDs.^33^ Research indicates that graphene enhances electron transport by providing a percolating network, while CNTs improve mechanical strength, distributing stress during cycling.^35^ This combination outperforms traditional electrodes, offering a scalable approach to high-performance energy storage. The composites also enhance thermal stability, crucial for long-term operation under varying conditions.^33^ Unlike static bulk materials, these hybrids balance energy and power density, addressing key challenges in both battery and supercapacitor technologies. Their adaptability stems from the complementary properties of QDs and conductive matrices, enabling efficient ion and electron pathways.^39^ This positions CdSe QD composites as a promising solution for advanced systems requiring durability and efficiency, from portable electronics to large-scale energy storage applications.

Integrating CdSe QDs with conductive matrices such as graphene, CNTs, or conductive polymers (*e.g.*, polyaniline or PEDOT:PSS) creates hybrid structures with enhanced electrochemical and mechanical stability, critical for high-performance batteries and supercapacitors. The primary mechanism involves the formation of a percolating conductive network that overcomes the low intrinsic electron mobility of standalone CdSe QDs (1–50 cm^2^ V^−1^ s^−1^).^36^ For instance, in LIBs, graphene–CdSe composites achieve specific capacities of ∼800 mA h g^−1^ by providing a high-conductivity scaffold (mobility >1000 cm^2^ V^−1^ s^−1^) that shortens Li^+^ diffusion pathways and reduces charge transfer resistance.^33^ This is driven by π–π interactions and van der Waals forces anchoring QDs to graphene's surface, ensuring uniform dispersion and minimizing agglomeration during cycling. In supercapacitors, CNT–CdSe hybrids deliver specific capacitances of ∼600 F g^−1^, leveraging CNTs' tubular structure to facilitate rapid electron transfer and ion accessibility, enhancing pseudocapacitive efficiency.^35^ The high surface area of CNTs (∼500 m^2^ g^−1^) complements CdSe's nanoscale dimensions, increasing active sites for ion adsorption and faradaic reactions.^36^ Conductive polymers, such as polyaniline, further enhance stability by encapsulating CdSe QDs, buffering volume expansion during charge–discharge cycles and mitigating mechanical stress, which is critical for maintaining electrode integrity in sodium-ion batteries (80% capacity retention after 500 cycles).^27^ Mechanically, these matrices distribute stress evenly across the hybrid structure, preventing QD pulverization or detachment, a common issue in conversion-type electrodes. Additionally, the robust framework reduces strain from ion intercalation, as evidenced by TEM studies showing intact QD–matrix interfaces after 1000 cycles.^70^ These synergistic effects—improved conductivity, enhanced ion diffusion, and reinforced mechanical stability—position CdSe-based hybrids as superior to traditional electrodes, enabling high-rate performance and long-term durability in energy storage applications.

To elucidate the performance of CdSe QD-based hybrid composites relative to benchmark electrode materials, Table 3 provides a quantitative comparison across specific capacity/capacitance, cycling stability, and energy efficiency under practical energy storage conditions. This comparison highlights the advantages and limitations of CdSe QD hybrids in LIBs and supercapacitors, contextualizing their potential for applications like electric vehicles and grid storage. This comparison demonstrates that CdSe QD hybrids offer superior specific capacity/capacitance and high-rate performance compared to graphite and activated carbon, but their cycling stability lags behind due to nanoscale-induced volume changes and defect formation. Energy efficiency is competitive, particularly with photo-assisted enhancements, but cost and scalability challenges limit commercial adoption. These metrics underscore the need for optimized synthesis and cadmium-free alternatives to bridge the gap with benchmark materials.

**Table 3:** Comparative Performance of CdSe QD hybrids *vs.* benchmark electrode materials

Material	Application	Specific capacity/capacitance	Cycling stability	Energy efficiency	Remarks	Ref.
Graphene–CdSe QD	LIBs	800 mA h g^−1^ (0.5C)	80–85% retention after 500 cycles	85–90%	High capacity, but limited by volume expansion	33
Graphite	LIBs	372 mA h g^−1^ (0.5C)	95% retention after 1000 cycles	90–95%	Industry standard, excellent stability	59
LiCoO_2_	LIBs	550 mA h g^−1^ (0.5C)	90% retention after 500 cycles	85–90%	High stability, but lower rate capability	14
CNT–CdSe QD	Supercapacitors	600 F g^−1^	90% retention after 10 000 cycles	∼90%	Enhanced pseudocapacitance, high-rate performance	35
Activated carbon	Supercapacitors	500–600 F g^−1^	95% retention after 10 000 cycles	∼95%	Cost-effective, excellent durability	17
MnO_2_	Supercapacitors	∼1000 F g^−1^	80% retention after 10 000 cycles	∼85%	High capacitance, but limited stability	18

### 6.5. Structural stability

CdSe QDs' nanoscale size (2–10 nm) enhances structural stability by minimizing volume expansion during charge–discharge cycles, a common failure mode in conversion-type electrodes. In sodium-ion batteries, they retain 80% capacity after 500 cycles,^34^ as their uniform distribution reduces mechanical stress compared to bulk materials. This durability is essential for long-term applications like EVs or stationary storage, where consistent performance over extended periods is critical. The small size limits pulverization, preserving electrode integrity during repeated cycling.^27^ In supercapacitors, CdSe QDs maintain 90% capacitance after 10 000 cycles, resisting degradation under rapid charge–discharge conditions. This resilience suits high-power uses like regenerative braking, where electrodes face intense mechanical and electrochemical stress.^70^ The nanoscale structure distributes strain evenly, preventing cracking seen in larger particles, and ensures sustained electrochemical activity.^27^ Studies show that smaller QDs exhibit greater resistance to volume changes due to their high surface-to-volume ratio, stabilizing active sites over time.^71^ This contrasts with bulk electrodes, which degrade faster under similar conditions, highlighting CdSe QDs' advantage in longevity. Integration into composites further enhances stability by providing a supportive framework, reducing strain and improving cycle life. The ability to maintain structural integrity under redox and ion intercalation processes makes CdSe QDs a robust material for energy storage. Their stability addresses a key challenge in both high-energy batteries and high-power supercapacitors, ensuring reliable operation over thousands of cycles. This positions them as a critical component in next-generation devices, where mechanical durability and electrochemical performance must coexist for practical deployment in demanding applications.

### 6.6. Photoelectrochemical effects

CdSe QDs' strong light absorption (460–660 nm) generates electron–hole pairs, enhancing charge storage under illumination and enabling photoelectrochemical applications. In batteries, this increases capacity by 20%, integrating solar energy harvesting with storage for self-powered devices like sensors or wearable's. The size-tunable absorption optimizes photon capture, amplifying charge availability during cycling.^25^ Smaller QDs enhance stability under illumination, while larger ones maximize light harvesting; tailoring performance to specific needs. In supercapacitors, photocapacitive effects boost capacitance by 15%,^55^ supporting renewable energy systems like solar-powered electronics. This optoelectronic synergy leverages quantum-confined properties, distinguishing CdSe QDs from traditional materials reliant solely on electrical input. The photo-induced boost reduces overpotentials in batteries, improving cycle life by facilitating oxygen reactions or Li^+^ intercalation.^25^ In supercapacitors, it enhances charge separation, increasing pseudocapacitive efficiency under pulsed conditions.^55^ Studies show that CdSe QDs' absorption spectra shift with size, enabling precise control over photoelectrochemical responses.^25^ This capability is particularly promising for hybrid solar-storage devices, where energy generation and storage occur seamlessly, and addressing intermittency in renewable sources. The mechanism relies on efficient exciton generation and transfer, driven by the QDs' high photoluminescence quantum yield. Unlike conventional electrodes, CdSe QDs couple optical and electrochemical properties, offering a transformative approach to sustainable energy storage. Their ability to enhance charge storage under light positions them as a key material for next-generation systems, from photo-rechargeable batteries to photocapacitors, where dual energy input improves overall efficiency and performance in environmentally friendly applications.

### 6.7. Defect engineering

Surface passivation of CdSe QDs (*e.g.*, with ZnS) reduces defect-induced trap states, enhancing electrochemical stability in energy storage devices. In batteries, this improves Li^+^ intercalation efficiency by minimizing non-radiative recombination, as shown in studies where passivated QDs enhance charge storage dynamics. The high surface area of QDs (2–10 nm) increases defect susceptibility, but passivation neutralizes these sites, boosting radiative recombination and sustaining charge carrier generation. This is critical for LIBs, where stable ion diffusion pathways improve capacity retention over cycles.^54^ In supercapacitors, defect engineering minimizes charge recombination, stabilizing pseudocapacitance and maintaining performance, with passivated CdSe QDs achieving consistent redox activity in graphene composites.^70^ The passivation layer (*e.g.*, ZnS) enhances photoluminescence quantum yield, reducing energy losses under electrochemical stress. Unlike unpassivated QDs, where carrier trapping hampers efficiency, passivated structures rival traditional materials prone to degradation. Research indicates that ZnS shells increase active sites for ion interactions without compromising structural integrity, improving long-term cycling stability. This approach leverages the QDs' nanoscale properties, tailoring them for rigorous demands in energy storage.^54^ In batteries, it supports high-rate applications by ensuring smooth charge transfer, while in supercapacitors, it enhances power delivery by maintaining redox efficiency.^68^ The reduction in surface traps also improves environmental stability, protecting QDs from oxidative damage during operation. This positions CdSe QDs as a robust alternative for advancing both battery and supercapacitor performance, particularly in systems requiring durability and efficiency, such as EVs or renewable energy grids, where defect management is key to sustained electrochemical excellence. Table 4 displays comparative electrochemical mechanisms of CdSe QDs in batteries and supercapacitors.

**Table 4:** Comparative electrochemical mechanisms of CdSe QDs in batteries and supercapacitors

Mechanism	Batteries	Supercapacitors
Surface area and charge dynamics	High surface area (>100 m^2^ g^−1^, 2–10 nm) and fast electron transfer (0.5–7 ns TRPL) enable Li^+^ intercalation; *e.g.*, 700 mA h g^−1^ at 10C	Enhances EDLC *via* rapid ion adsorption; *e.g.*, 400 F g^−1^ with 10 kW kg^−1^ power density
Pseudocapacitance	Faradaic redox (Cd^2+^/Se^2−^) and conversion (CdSe + 2Li^+^ → Cd + Li_2_Se) yield 500–900 mA h g^−1^	Surface redox boosts capacitance; *e.g.*, 550 F g^−1^ in graphene–CdSe composites
Tunable bandgap	Size-tuned bandgap (1.7–2.5 eV) enhances conductivity and rate capability in LIBs	Optimizes charge transfer kinetics; *e.g.*, 1.7 eV boosts rate performance
Synergistic composites	Graphene–CdSe hybrids improve conductivity and stability; *e.g.*, 800 mA h g^−1^ in LIBs	Enhances kinetics and retention; *e.g.*, 600 F g^−1^ in graphene–CdSe composites
Structural stability	Mitigates volume expansion; *e.g.*, 80% retention after 500 cycles in Na-ion batteries	Maintains integrity; *e.g.*, 90% capacitance after 10 000 cycles
Photoelectrochemical effects	Light-induced charge generation boosts capacity by 20% (460–660 nm absorption)	Photocapacitive enhancement of 15% in capacitance
Defect engineering	ZnS passivation reduces traps, enhancing Li^+^ intercalation efficiency	Minimizes charge recombination, stabilizing pseudocapacitance

### 6.8. Quantum confinement and long-term electrochemical stability

The quantum confinement effect in CdSe QDs, driven by their nanoscale size (2–10 nm), significantly influences long-term electrochemical stability by modulating electronic properties and structural integrity during repeated charge–discharge cycles. The size-dependent bandgap (1.7–2.5 eV) enhances electrochemical stability by tailoring redox potentials to minimize side reactions with electrolytes, a common degradation mechanism in LIBs. Smaller QDs with wider bandgaps (*e.g.*, 2.5 eV) exhibit greater resistance to oxidative degradation, maintaining stable Li^+^ intercalation efficiency over 500 cycles with 80% capacity retention in sodium-ion batteries.^27^ This stability stems from quantum confinement's ability to confine charge carriers, reducing non-radiative recombination and preserving electronic integrity under electrochemical stress.^54^ Structurally, the nanoscale dimensions of CdSe QDs mitigate volume expansion during ion intercalation, a key factor in preventing electrode pulverization. For instance, conversion reactions (CdSe + 2Li^+^ → Cd + Li_2_Se) in LIBs induce less mechanical strain in QDs compared to bulk materials, as their high surface-to-volume ratio distributes stress evenly, preserving lattice integrity.^61^ In supercapacitors, quantum confinement enhances pseudocapacitive stability by increasing the availability of surface redox sites, with CdSe QDs retaining 90% capacitance after 10 000 cycles.^70^ However, repeated cycling can introduce surface defects, slightly widening the bandgap and reducing conductivity over time, as observed in TRPL studies showing increased decay times after 1000 cycles.^24^ Surface passivation with ZnS shells mitigates this by neutralizing trap states, ensuring sustained electrochemical performance.^23^ These mechanisms highlight how quantum confinement bolsters CdSe QDs' resilience, enabling them to maintain structural and electronic stability under rigorous cycling conditions, making them ideal for long-term energy storage applications such as electric vehicles and renewable energy grids.

## 7. Challenges, advancements, and future potential of CdSe QDs in batteries and supercapacitors

### 7.1. Challenges of CdSe QDs in energy storage

CdSe QDs offer a compelling platform for energy storage, yet their integration into practical systems like batteries and supercapacitors is fraught with operational and material-specific challenges that demand innovative solutions. One pressing issue is their susceptibility to thermal degradation under demanding conditions, such as the rapid charge–discharge cycles required in electric vehicle (EV) applications. At the nanoscale, CdSe's melting point drops significantly below that of its bulk form due to increased surface energy, making it prone to structural changes when subjected to localized heating during high-rate operation.^44^ This thermal instability can trigger phase transitions or particle coalescence, compromising the electrochemical integrity of electrodes over extended cycling. Unlike conventional materials like graphite, which maintain stability under similar stresses, CdSe QDs require sophisticated thermal management strategies—such as advanced cooling or heat-dissipating composites—to ensure reliability in power-intensive scenarios. Electrochemical compatibility with electrolytes presents another formidable hurdle. In LIBs, CdSe QDs can interact unfavorably with standard electrolytes, forming insulating surface layers that hinder ion transport and increase impedance over time.^63^ To address electrochemical incompatibility and degradation of CdSe QDs in energy storage systems, various electrolyte engineering strategies have been developed to enhance stability and performance. Table 5 summarizes key approaches, including ionic liquids, protective coatings, and electrolyte additives, which have shown success in minimizing QD degradation in LIBs and supercapacitors. These strategies mitigate issues such as insulating surface layer formation, selenium dissolution, and oxidative degradation, improving long-term cycling stability and electrochemical efficiency.

**Table 5:** Electrolyte engineering strategies for minimizing CdSe QD degradation in energy storage systems

Strategy	Description	Application	Effectiveness	Challenges	Ref.
Ionic liquids	Use of non-volatile, thermally stable ionic liquids (*e.g.*, [BMIM][PF6])	LIBs, supercapacitors	Reduces selenium dissolution by 50% in aqueous electrolytes; enhances cycling stability (90% retention after 1000 cycles)	High cost, lower ionic conductivity	94
Protective coatings	Application of ZnS or SiO_2_ shells (∼5–10 nm) to CdSe QDs	LIBs, supercapacitors	Prevents insulating layer formation; retains 85% PL intensity after 1000 cycles	Increased synthesis complexity, cost	23 and 56
Electrolyte additives	Incorporation of vinylene carbonate (VC) or fluoroethylene carbonate (FEC)	LIBs	Suppresses side reactions; improves capacity retention by 10–15% after 500 cycles	Limited effect in aqueous supercapacitors	95
Polymer electrolytes	Gel electrolytes (*e.g.*, PMMA-based) to encapsulate QDs	Supercapacitors	Enhances mechanical stability; reduces corrosion by 40% in aqueous systems	Reduced ion mobility in gel matrices	96
Hybrid electrolytes	Combination of organic solvents and ionic liquids (*e.g.*, EC/DMC with [EMIM][TFSI])	LIBs	Minimizes oxidative degradation; improves rate capability by 20%	Complex formulation, higher viscosity	97

CdSe QDs offer significant advantages for energy storage due to their tunable electronic properties, high surface area, and enhanced charge transport, enabling high specific capacities (*e.g.*, ∼600 mA h g^−1^ in LIBs) and pseudocapacitive behavior (*e.g.*, 550 F g^−1^ in supercapacitors).^70^ However, their practical viability is hindered by critical challenges, including cadmium toxicity, which poses health and environmental risks due to its carcinogenic nature, necessitating stringent disposal protocols. Long-term stability is another concern, as CdSe QDs often suffer from surface oxidation and electrolyte incompatibility, leading to capacity fade after ∼500 cycles in LIBs.^63^ Scalability issues, such as the high cost and complexity of hot injection synthesis, further limit industrial adoption, with production costs estimated at 2–3 times higher than carbon-based nanomaterials.^94^ In comparison, lead sulfide (PbS) QDs offer similar tunability but face comparable toxicity issues, while indium phosphide (InP) QDs are less toxic but exhibit lower electrochemical performance (∼300 mA h g^−1^) due to weaker quantum confinement.^98^ Non-QD nanomaterials, such as graphene and transition metal oxides (*e.g.*, MnO_2_), provide higher stability and lower toxicity but lack the precise bandgap control of CdSe QDs, limiting their versatility in hybrid systems.^99^ This comparison underscores the need for greener synthesis methods, non-toxic QD alternatives, and hybrid designs to balance performance and practicality for large-scale energy storage applications.

These reactions stem from the redox activity of cadmium and selenium, which can catalyze side reactions with electrolyte components, degrading both the QD surface and the electrolyte itself. In supercapacitors, aqueous electrolytes pose a risk of corrosion, as selenium tends to dissolve under prolonged exposure, while organic alternatives introduce safety concerns and lower ionic conductivity.^35^ This mismatch necessitates the development of tailored electrolytes or protective coatings that can stabilize CdSe without sacrificing performance, a task complicated by the need to balance chemical stability with ionic accessibility. Fabrication challenges further complicate CdSe QD deployment, particularly in achieving uniform dispersion within electrode structures. The strong interparticle forces at play in nanoscale systems lead to agglomeration during processing or cycling, disrupting the homogeneity critical for efficient charge transfers.^80^ This clustering creates regions of inconsistent electrochemical activity, undermining the uniformity that large-scale energy storage devices demand. While surfactants or dispersants can mitigate this, their degradation under operational conditions releases byproducts that destabilize the electrode environment.^53^ Unlike carbon-based materials, which benefit from well-established dispersion techniques, CdSe QDs require novel fabrication approaches—potentially involving advanced mixing or templating methods—to maintain their nanoscale advantages in practical electrodes. Interfacial charge transfer kinetics also pose a significant barrier, especially in hybrid systems integrating CdSe QDs with conductive scaffolds like graphene or carbon nanotubes (CNTs). The electronic mismatch between CdSe's tunable bandgap and the fixed properties of carbon matrices creates energy barriers at their junctions, slowing electron movement and reducing overall efficiency.^63^ This is particularly pronounced in supercapacitors, where rapid charge–discharge is paramount, and in LIBs under high-rate conditions, where sluggish kinetics limit responsiveness.^61^ Surface modifications, such as thin insulating shells, can enhance conductivity, but their application must be precisely controlled to avoid compromising ion access or mechanical stability during volume changes inherent to cycling.^16^ Optimizing these interfaces demands a deeper understanding of heterojunction dynamics and tailored engineering to align CdSe's properties with those of its host materials.

The interplay between nanoscale-induced electron mobility constraints in CdSe QDs and their hybridization with conductive matrices like graphene or CNTs significantly influences the scalability, long-term cycling stability, cost-effectiveness, and environmental sustainability of hybrid energy storage systems. CdSe QDs' low intrinsic electron mobility (1–50 cm^2^ V^−1^ s^−1^) due to surface traps limits charge transfer efficiency, necessitating hybridization with high-mobility matrices (*e.g.*, graphene: >1000 cm^2^ V^−1^ s^−1^) to achieve specific capacities of ∼800 mA h g^−1^ in LIBs and capacitances of ∼600 F g^−1^ in supercapacitors.^33,35^ This synergy enhances cycling stability (80–90% capacity retention after 1000 cycles) by forming a percolating conductive network that mitigates QD agglomeration and distributes mechanical stress during ion intercalation.^70^ However, scalability is hindered by the complex synthesis of uniform QDs (*e.g.*, hot-injection at 300–350 °C) and the high cost of graphene/CNT production, which elevate electrode costs compared to graphite anodes.^24^ Long-term cycling stability, while improved, remains inferior to silicon anodes (90% retention after 1000 cycles, 3000 mA h g^−1^) due to CdSe's susceptibility to defect formation over extended cycles.^61^ Cost-effectiveness is further challenged by cadmium's toxicity, classified as carcinogenic under RoHS and EPA regulations, requiring costly ZnS encapsulation and recycling processes, unlike non-toxic solid-state electrolytes with simpler disposal.^23^ Environmentally, CdSe QD hybrids lag behind lithium–sulfur batteries, which avoid heavy metals and achieve similar energy density (∼500 W h kg^−1^) with lower lifecycle impacts.^22^ Compared to silicon, CdSe hybrids offer faster charge transfer but face stricter regulatory hurdles, limiting adoption in cost-sensitive applications like grid storage. To enhance feasibility, cadmium-free alternatives like ZnSe QDs (specific capacitances ∼400 F g^−1^) and scalable microwave-assisted synthesis are being explored to reduce costs and environmental impact.^48^ These trades-offs highlight that while CdSe QD hybrids excel in high-rate performance, their scalability and sustainability require significant advancements to compete with mature and emerging technologies in real-world energy storage applications.

Beyond technical hurdles, CdSe QDs face regulatory and economic constraints that impact their viability. Cadmium's status as a hazardous substance under global regulations imposes stringent handling, disposal, and recycling requirements, elevating lifecycle costs and complicating supply chains.^1^ Compared to established materials like graphite or activated carbon, which benefit from mature production ecosystems, CdSe QDs remain cost-prohibitive for widespread adoption, particularly in price-sensitive applications like grid storage.^100^ Recycling poses an additional challenge, as separating cadmium from spent electrodes is less efficient than recovering metals from conventional systems, raising sustainability concerns.^37^ Addressing these issues requires not just material innovation but also strategic efforts to navigate regulatory landscapes and develop cost-effective production pathways, ensuring CdSe QDs can compete in a market dominated by cheaper, less restricted alternatives.

The toxicity of cadmium in CdSe QDs poses significant environmental and health risks, limiting their widespread commercial adoption in energy storage applications. Cadmium is classified as a carcinogenic and hazardous substance under global regulations, such as the European Union's Restriction of Hazardous Substances (RoHS) directive and the U.S. Environmental Protection Agency (EPA) guidelines, imposing strict handling, disposal, and recycling requirements. These regulations increase lifecycle costs, as separating cadmium from spent electrodes is less efficient than recovering materials like graphite or lithium, complicating supply chains and raising sustainability concerns.^1^ Practical limitations include the high cost of safe manufacturing and disposal processes, which can render CdSe QDs less competitive compared to established materials like activated carbon or metal oxides in cost-sensitive applications such as grid storage. Additionally, the risk of cadmium leaching during device operation or disposal necessitates robust encapsulation, such as ZnS or silica shells, which adds complexity and cost to production.^23^ To address these challenges, research has advanced cadmium-free alternatives like ZnSe and CuInS_2_ QDs, which retain comparable electrochemical properties (*e.g.*, tunable bandgaps of 2.0–2.7 eV and specific capacitances of ∼400 F g^−1^) while aligning with environmental regulations.^24^ Furthermore, recycling innovations, such as hydrometallurgical processes tailored for QD recovery, are being explored to enhance sustainability. These strategies mitigate regulatory barriers, but their implementation requires significant investment, underscoring the need for cost-effective synthesis and lifecycle management to enable CdSe QDs' commercial viability in energy storage systems.

### 7.2. Advancements in CdSe QDs for enhanced performance

Recent progress in CdSe QD research has yielded transformative advancements, harnessing their nanoscale properties to elevate performance in energy storage systems. A key breakthrough lies in their ability to enhance charge storage, driven by their expansive surface area and quantum confinement effects. In LIBs, CdSe QDs integrated with graphene outperform traditional anodes by leveraging short ion diffusion paths and efficient redox reactions (CdSe + 2Li^+^ → Cd + Li_2_Se), offering superior capacity and responsiveness under varying conditions.^64^ In supercapacitors, they amplify both electric double-layer and pseudocapacitive contributions, delivering rapid energy delivery that surpasses carbon-based electrodes, thanks to their ability to store charge across their extensive surfaces.^35^ This dual functionality stems from the interplay of size-dependent electronic states; allowing CdSe to adapt to diverse electrochemical demands. Surface engineering has emerged as a cornerstone of these advancements, with passivation techniques significantly reducing performance-limiting defects. Coatings like ZnS or silica neutralize surface trap states, enhancing charge retention and stability during cycling.^23^ In photo-assisted systems, such as Li–O_2_ batteries, these passivated QDs lower reaction barriers, facilitating oxygen evolution and reduction with improved efficiency and longevity.^27^ The protective layers also shield CdSe from environmental degradation, maintaining electrochemical activity over prolonged operation—a critical factor for supercapacitors requiring consistent performance across thousands of cycles.^40^ This approach transforms a once-vulnerable material into a robust contender, aligning its durability with practical energy storage needs.

The tunability of CdSe's bandgap stands out as a versatile advantage, enabling precise optimization of electrochemical behavior. By adjusting QD size, researchers can shift electronic properties to favor either high-rate stability or enhanced conductivity, tailoring performance for specific applications. Smaller QDs excel in fast-charging scenarios, where rapid ion exchange is key, while larger ones boost charge transport in systems prioritizing power output.^60^ This flexibility, observable through shifts in optical absorption, allows CdSe to meet the distinct requirements of LIBs, supercapacitors, and beyond, offering a level of customization unmatched by bulk materials.^71^ Such adaptability positions CdSe as a dynamic building block for next-generation devices. Hybridization with conductive frameworks like graphene or CNTs has further elevated CdSe's capabilities, overcoming its inherent mobility constraints. These composites create synergistic networks that enhance electron transport and mechanical resilience, ensuring stable performance over extended cycling.^36^ In LIBs, the robust scaffold mitigates strain from volume changes, while in supercapacitors, it supports high-rate charge transfer, maintaining efficiency under stress. Advanced synthesis methods, such as microwave-assisted techniques, complement this by producing uniform QDs quickly and efficiently, reducing energy inputs and enhancing scalability.^48^ Together, these innovations bridge the gap between CdSe's nanoscale potential and industrial applicability.

Efforts to develop cadmium-free alternatives like ZnSe and CuInS_2_ have also gained traction, replicating CdSe's strengths with reduced environmental impact. These substitutes retain tunable electronic properties and high surface activity, performing admirably in both batteries and supercapacitors while aligning with sustainability goals.^24^ By combining these material advances with improved fabrication and integration strategies, CdSe QDs and their derivatives are evolving into versatile, high-performance options, poised to meet the rigorous demands of modern energy storage applications—from EVs to renewable grids.

### 7.3. Future potential and emerging directions

The future of CdSe QDs in energy storage is brimming with potential, promising to redefine performance benchmarks across batteries and supercapacitors. In LIBs, their integration into advanced composites could push charge storage beyond current limits, supporting longer-range EVs and more efficient grid systems.^61^ For sodium-ion batteries (SIBs), CdSe offers a pathway to affordable, high-capacity storage, leveraging its redox versatility to outperform traditional materials in cost-sensitive applications. Solid-state batteries stand to gain from CdSe's surface properties, which could enhance electrolyte interfaces and enable faster charging, critical for next-generation EV adoption.^88^ In supercapacitors, CdSe's ability to combine rapid charge delivery with pseudocapacitive boosts positions it as a candidate for high-power uses, from regenerative braking to stabilizing renewable energy outputs.^35^ A particularly exciting direction is the development of solar-storage hybrids, where CdSe's photoelectrochemical capabilities shine. By absorbing light across a broad spectrum, these QDs could enable devices that simultaneously generate and store energy, powering autonomous sensors or wearables with minimal external input.^25^ Flexible, photo-responsive systems integrating CdSe with related compounds like CdS demonstrate mechanical durability alongside energy conversion, opening doors to wearable electronics and portable power. Such hybrids could streamline renewable energy infrastructure by addressing intermittency, merging generation and storage into a single, efficient unit.^55^

Sustainability drives another key avenue, with cadmium-free QD variants like ZnSe and CuInS_2_ poised to expand CdSe's legacy. These alternatives replicate its electrochemical strengths—tunable bandgaps and high surface activity—while sidestepping regulatory and environmental barriers, making them viable for widespread adoption.^24^ Scalable synthesis techniques, such as rapid microwave methods, further support this transition, producing consistent QDs with lower resource demands, aligning with industrial needs for cost-effective production.^48^ This shift could democratize QD-based energy storage, extending its reach from niche applications to mainstream markets like grid-scale renewables and consumer devices.

Beyond conventional uses, CdSe QDs hold promise for specialized applications, such as high-power pulsed systems in aerospace or medical technology, where their rapid kinetics enable burst energy delivery.^71^ Their incorporation into additive manufacturing—like 3D-printed electrodes—could yield customized storage solutions, blending flexibility with performance for emerging tech.^35^ As research deepens, CdSe QDs could spearhead a new era of energy storage, marrying high efficiency, environmental consciousness, and innovative design to meet global energy challenges.

## 8. Conclusion

CdSe QDs hold transformative potential for energy storage, yet their integration into batteries and supercapacitors faces significant hurdles. This review explores the operational challenges limiting CdSe QDs, including thermal instability under high-rate conditions, electrochemical incompatibility with electrolytes, and difficulties in achieving uniform electrode dispersion. These issues, compounded by regulatory constraints due to cadmium's toxicity, necessitate innovative solutions to unlock their full potential. Recent advancements have bolstered CdSe's viability, leveraging surface passivation with coatings like ZnS to enhance stability, bandgap tunability to optimize performance, and hybridization with conductive matrices such as graphene to improve charge transfer and scalability. These developments enable CdSe QDs to outperform conventional materials in charge storage and rapid energy delivery, with cadmium-free alternatives like ZnSe emerging as sustainable options. Looking ahead, CdSe QDs promise to revolutionize lithium-ion and solid-state batteries, supercapacitors, and solar-storage hybrids by combining high efficiency with photoelectrochemical capabilities. Their adaptability also positions them for specialized applications, from pulsed power systems to 3D-printed electrodes. This review underscores CdSe QDs' trajectory toward practical, high-performance energy storage, balancing technical innovation with environmental and economic considerations.

## Data availability

This article is a review that synthesizes and analyzes existing research on cadmium selenide (CdSe) quantum dots (QDs) for batteries and supercapacitors. No new experimental data were generated or analyzed in this study. All data, figures, and information presented are derived from previously published studies, which are cited throughout the manuscript and listed in the References section. The original data supporting the findings discussed herein are available in the cited publications, and readers are directed to those sources for further details.

## Conflicts of interest

There are no conflicts to declare.
